# Effectiveness of a self-management intervention on clinical outcomes among patients with type 2 diabetes at high risk for Diabetic Kidney Disease: A Randomised Controlled Trial

**DOI:** 10.1371/journal.pone.0360475

**Published:** 2026-10-12

**Authors:** Dongmei Chu, Bingyang Liu, Kim Lam Soh, Hasni Idayu Saidi, Salimah Japar

**Affiliations:** 1 Department of Nursing, Faculty of Medicine and Health Sciences, Universiti Putra Malaysia, Selangor, Malaysia; 2 Department of Biomedical Sciences, Faculty of Medicine and Health Sciences, Universiti Putra Malaysia, Selangor, Malaysia; University of Pisa, ITALY

## Abstract

**Background:**

Diabetic kidney disease (DKD) is a common complication of type 2 diabetes mellitus (T2DM) and a leading cause of end-stage renal disease, posing substantial health and economic burdens. Early self-management interventions targeting modifiable risk factors are crucial to prevent or slow DKD progression. However, most studies focus on later stages of DKD, leaving a gap in early prevention for high risk of DKD individuals.

**Objective:**

To evaluate the effectiveness of a structured self-management intervention on clinical outcomes among T2DM patients at high risk of DKD.

**Design:**

A prospective, two-arm, randomized controlled trial.

**Setting:**

A tertiary hospital in Shandong Province, China.

**Participants:**

134 patients with T2DM who were classified as being at high risk of DKD using a validated DKD risk prediction model.

**Methods:**

134 participants were randomized 1:1 to the intervention or control group. The intervention group received a 12-week a structured self-management program and 12-week follow-up. The control group received usual care. Clinical outcomes included hemoglobin A1c (HbA1c), urinary albumin-to-creatinine ratio (UACR), estimated glomerular filtration rate (eGFR), systolic blood pressure (SBP), low-density lipoprotein cholesterol (LDL-C), body mass index (BMI), waist circumference (WC) were collected at baseline (T0), after the 12-week intervention (T1), and at 24 weeks (T2). Generalized estimating equations and intention-to-treat analyses were performed. Sensitivity analyses using multiple imputation were conducted.

**Results:**

All participants completed the intervention phase. During the follow-up visits period, 7 participants in the intervention group and 9 participants in the control group were lost to follow-up at T1 (attrition rate: 11.94%), while 5 participants in the intervention group and 4 participants in the control group were lost to follow-up at T2 (attrition rate: 6.71%). No significant differences in baseline sociodemographic or clinical indicators between participants retained and those lost to follow-up (p > 0.05), suggesting data were missing at random. Baseline comparisons between the intervention and control groups revealed no significant differences in demographic or clinical indicators (p > 0.05). Compared with the control group, the intervention group demonstrated significant improvements in HbA1c, eGFR, SBP, and LDL-C, while no significant differences were observed for UACR, BMI, or WC. Sensitivity analyses yielded consistent results.

**Conclusions:**

The self-management intervention effectively improved HbA1c, eGFR, SBP and LDL-C among T2DM patients at high risk of DKD. These findings suggest that integrating structured self-management into routine diabetes care may help improve modifiable risk factors and support early DKD prevention.

**Registration:**

China Clinical Trial Registry (ChiCTR2400092745; registered on 22 November 2024)

## 1. Background

The 10th edition of the IDF Diabetes Atlas confirms that Diabetes Mellitus (DM) is one of the fastest-growing global health emergencies of the 21st century, it is estimated that 537 million people with diabetes in 2021, this number is projected to reach 643 million by 2030 and 783 million by 2045 [1,2]. The rising prevalence has a significant implications for Diabetic Kidney Disease (DKD). DKD is one of the most common microvascular complication and it is the leading cause of end-stage renal disease (ESRD) [3,4]. Current studies reported 20.0% to 40.0% of diabetic patients develop DKD [5,6]. González-Pérez et al. [7] reported the all-cause mortality rate for DKD is 50.3 per 1000 person-years, with cardiovascular disease (CVD) mortality rate at 8.0, and ESRD-related mortality rate at 6.9, posing a severe threat to the lives of DKD individuals.

Clinically, DKD is primarily evaluated using the urinary albumin-to-creatinine ratio (UACR) and the estimated glomerular filtration rate (eGFR). UACR levels ≥30 mg/g are considered indicative of microalbuminuria, representing the early stage of overt renal damage. eGFR provides an objective measure of renal function and can be used to monitor changes over time [5,8].

Previous studies have explored various risk factors associated with DKD progression in patients with T2DM, including sociodemographic factors, lifestyle factors and clinical indicators [9–14]. These factors can be categorized as non-modifiable and modifiable factors. Non-modifiable factors, such as age, gender, genetic susceptibility and modifiable factors, including glycemia, hypertension, dyslipidemia and obesity, which can be regulated through lifestyle modification and self-management. Poor glycemic control induces glomerular hyperfiltration, oxidative stress, and renal inflammation; elevated blood pressure increases intraglomerular pressure and accelerates proteinuria and renal function decline; dyslipidemia promotes lipid accumulation and endothelial dysfunction; while excess body weight, particularly central fat, exacerbates insulin resistance and systemic inflammation, further aggravating renal damage, these metabolic together forming key biological pathways driving DKD progresses [15–17].

Considering the severity and far-reaching impact of DKD, clinical guideline for the prevention and treatment of DKD emphasize early screening, early diagnosis, early treatment, and integrated comprehensive intervention [18]. In particular, strict control of hyperglycemia and hypertension has been recognized as a cornerstone in delaying the progression of DKD [19]. Therefore, self-management is particularly important, as it enables patients to actively participate in controlling these modifiable risk factors.

Self-management refers to an individual’s ability to take an active role in their own care and well-being, making informed decisions and adopting behaviors that promote health and manage chronic conditions. By actively participating in self-management, individuals can better control their own health, improve their quality of life, and reduce the risk of disease. American Diabetes Association Professional Practice Committee [20] emphasizes the crucial role of self-management education for diabetic patients in establishing positive health behaviors and maintain mental well-being. Kidney Disease: Improving Global Outcomes (KDIGO) Diabetes Work Group [21] also recommend that a structured self-management educational program be implemented for care of people with diabetes and chronic kidney disease.

Patients who are skilled in self-management and actively participating in disease management can reduce modifiable risk factors, thereby have improved health outcomes. Over the past years, diabetes self-management interventions have been shown to be effective and cost-effective in promotion and facilitation of self-management, with improvements in patients’ knowledge, skills, and motivation leading to improved biomedical, glycemic control, behavioral, and psychosocial outcomes [22–24].

Currently, self-management interventions for DKD patients primarily focus on individuals with established disease, particularly those at the microalbuminuria or dialysis stages. However, given that renal damage is often irreversible, early intervention is particularly meaningful. Despite this, evidence on structured self-management interventions targeting patients at high risk of DKD remains limited, and such individuals are often overlooked in current clinical practice. By using a DKD risk prediction model [25], high risk individuals among T2DM patients can be identified for early intervention. This approach aims to enhance patients’ self-management behaviors and improve clinical indicators that serve as risk factors for the development of DKD, ultimately delay or mitigate DKD progression and complications.

### Objective

To evaluate the effectiveness of a structured self-management intervention on clinical outcomes among T2DM patients at high risk of DKD.

## 2. Methods

### 2.1 Study design and setting

The study design is a prospective, two-arm, assessor-blinded, randomized controlled trial. It has also been registered in the China Clinical Trial Registry on 22 November 2024, with registration number ChiCTR2400092745. The study is conducted at a tertiary hospital located in Shandong Province, China.

### 2.2 Inclusion Criteria and Exclusion Criteria

Patients were screened according to predefined inclusion and exclusion criteria. Inclusion criteria were as follows: (a) Patients meet the World Health Organization diagnostic criteria for T2DM; (b) Patients at high risk for DKD (DKD risk scores ≥16). (c) Age 39–75 years old; (d) Patients can use WeChat; (e) Voluntarily join the study and sign the informed consent form. Exclusion Criteria were (a) Patients with DKD; (b) Those who have mental disorders, cognitive impairment, etc. and are unable to cooperate with the researcher; (c) Other secondary glomerular diseases caused by primary renal disease and hypertension, renal arteriosclerosis; (d) There have been recent factors affecting urinary protein such as diabetic ketoacidosis, acute nephritis, and urinary tract infection.

### 2.3 Sample Size

The sample size was calculated based on expected changes in HbA1c reported in a previous intervention study [26], with a baseline of 7.79 ± 1.82 and post-intervention mean of 7.06 ± 0.89. The calculation was performed using the standard formula n = 2σ^2^(Z_1_ ₋ α/2 + Z_1_ ₋ β)^2^/ (µ_1_ − µ_2_)^2^. Based on a two-sided significance level of 0.05 and a power of 80%, the required sample size was estimated to be 60 participants per group. Allowing for a 10% dropout rate, the total sample size was adjusted to 134 participants.

### 2.4 Recruitment, randomization and blinding

The recruitment poster was placed at the nurses’ station to recruit participants. Potential participants who were interested in the study were evaluated by the nursing manager to determine whether they met the inclusion criteria. Those who met the criteria and provided written informed consent were enrolled in the study.

Randomization was conducted using a pre-generated sequence of 134 unique random integers corresponding to the enrollment sequence numbers. This sequence was prepared by an independent individual using an online random number generator. Each random number was placed in a kraft paper envelope labeled on the outside with its corresponding enrollment number. Eligible participants received the envelope matching their enrollment sequence, and the number inside the envelope determined group allocation. Odd numbers were assigned to the intervention group, while even numbers were assigned to the control group. Participant recruitment was conducted from 2 December 2024–28 February 2025.

This study was a single-blind randomized controlled trial. Blinding was not feasible for the researchers delivering the intervention or for the data analysts due to the nature of the intervention. To reduce potential bias, outcome assessors and participants were kept unaware of group assignments. During data collection, participants were identified using their enrollment numbers rather than their allocation.

### 2.5 Intervention Methods

#### 2.5.1 Control group.

Upon admission, the physician evaluates the patient’s condition through a detailed inquiry and relevant examinations, develops a treatment plan, and issues medical orders. Medical orders typically include intravenous injection therapy, medications, and health education. Health education is delivered verbally by the primary nurse and covers basic diabetes knowledge (types, common complications, normal glucose levels), recognition and management of hyperglycemia and hypoglycemia, dietary advice, reducing alcohol intake, and encouraging safe moderate physical activity. For patients using insulin, medication instructions and injection techniques are also provided.

#### 2.5.2 Intervention group.

**2.5.2.1 Intervention Team:** The intervention team consists of one endocrinologist, one nursing manager, two diabetes-specialized nurses, and one nursing researcher. The endocrinologist released the recruitment information, contacted participants, and provided overall study supervision. The nursing manager screened and enrolled eligible patients and distributed sealed envelopes that indicated the group allocation. The two diabetes-specialized nurses were responsible for data collection, while the nursing researcher implemented the intervention and conducted the statistical analysis. All members received standardize training before the enrollment.

**2.5.2.2 Intervention Content:** Participants in the intervention group received a structured self-management program covering five core topics: (1) DKD knowledge, (2) diet management, (3) physical activity, (4) medication management, and (5) self-monitoring. The content was developed based on current evidence and major clinical guidelines for diabetes and chronic kidney disease, ensuring consistency with best practices and enhancing methodological rigor [20,21,27–29].

There is often a significant gap between knowledge and action, and merely providing individuals with health knowledge is insufficient to promote behavior change. While many individuals may understand the correct practices, they often struggle to implement them in their daily lives. The Health Belief Model (HBM) addresses this gap by identifying the key factors that influence behavior. It offers a scientific framework and systematic approach to designing more effective interventions [30]. These five topics are structured based on the theoretical framework of the HBM, which aims to increase participants’ perceived severity and susceptibility to DKD, clarify the benefits of behavioral change, and strengthen self-efficacy to support sustainable self-management behaviors’

The intervention was delivered through six video-based sessions produced by the researcher, with scripts developed strictly according to authoritative guidelines and aligned with the HBM framework. The six sessions are summarized in Table 1.

**Table 1:** Overview of the Self-Management Intervention Sessions.

Sessions	Topic	Content
Session 1	DKD knowledge	(1) An overview of DKD; (2) The severity of DKD; (3) Risk factors for DKD; (4) DKD risk assessment; (5) Personalized goal setting; (6) Confidence building.
Session 2	Diet management- Part I	(1) The importance and benefits of diet management; (2) Nutritional Status Assessment; (3) Estimation and Distribution of Total Energy Intake; (4) Functions and Intake Principles of the Three Macronutrients; (5) Glycemic Index and Food Selection Techniques; (6) The “My Plate” Model and Balanced Eating Method.
Session 3	Diet management- Part II	(1) Scientific Eating Sequence; (2) Personalized Dietary Adjustment Combined with Preferences; (3) Barriers and Coping Strategies in Diabetes Diet Management; (4) Dietary Recording and Feedback.
Session 4	Physical Activity	(1) The multiple benefits and health impacts of exercise; (2) Populations unsuitable for exercise; (3) Principles for developing personalized exercise plans; (4) Safety considerations during exercise; (5) Scientific exercise management for older adults with diabetes; (6) Overcoming barriers to regular exercise; (7) Guided practice of the command version Baduanjin.
Session 5	Medication management	(1) The importance and benefits of adhering to prescribed antidiabetic medications; (2) Usage methods and adverse effects of drugs; (3) Standardized insulin use; (4) Barriers and coping strategies for medication adherence.
Session 6	Self-monitoring	(1) The importance and benefits of self-monitoring; (2) Items and methods of self-monitoring; (3) Recognition and management of hypoglycemia; (4) Perceived barriers in self-monitoring; (5) Cues to action for overcoming barriers.

#### 2.5.3 Intervention periods.

The intervention comprises two periods: a six-week initial learning phase and a subsequent strengthening phase. The initial learning phase included six weekly sessions, while the strengthening phase focused on consolidating the knowledge and skills acquired earlier.

After completing the intervention, participants in the intervention group underwent a 12-week follow-up. During this period, researchers conducted monthly telephone or WeChat calls to monitor adherence, support self-management practices, and encourage the maintenance of healthy behaviors. At the end of follow-up, participants were notified by phone to schedule a return visit.

### 2.6 Outcomes

Sociodemographic data: Patients’ sociodemographic characteristics were collected using a self-designed questionnaire. The variables included age, gender, ethnicity, marital status, educational level, monthly family income, residential status, family history of diabetes, duration of diabetes, treatment method, work intensity, history of hypertension, frequency of blood glucose monitoring.

And clinical outcomes: Clinical outcomes were obtained from patients’ medical records. These included hemoglobin A1c (HbA1c), urinary albumin-to-creatinine ratio (UACR), estimated glomerular filtration rate (eGFR), systolic blood pressure (SBP), low-density lipoprotein cholesterol (LDL-C), body mass index (BMI), waist circumference (WC).

### 2.7 Data Collection

Two researchers, who were not involved in group assignments, collected and recorded participants’ data using enrollment sequence numbers for identification. Data were collected at three time points: baseline (T0, after enrollment), post-intervention (T1, 12 weeks after enrollment), and at the end of follow-up (T2, 24 weeks after enrollment). Laboratory indicators were recorded from medical reports by the researchers.

### 2.8 Data Analysis

Data were analyzed using SPSS 29.0. Normality of continuous variables was assessed with the Shapiro–Wilk test. Normally distributed variables are presented as mean ± standard deviation, while non-normal variables are expressed as median (P25, P75). Categorical variables are reported as frequencies and percentages. Baseline comparisons between groups were performed using independent t tests for normally distributed continuous variables, Mann–Whitney U tests for non-normal variables, and chi-square or Fisher’s exact tests for categorical variables.

To evaluate the effectiveness of the intervention over time, generalized estimating equations (GEE) were applied to analyze repeated-measures data and to account for within-subject correlations. The models included group, time, and the group × time interaction term to examine differential changes between the intervention and control groups. An exchangeable working correlation structure was applied, and robust standard errors were used. A normal distribution with an identity link function was specified. For skewed variables, log-transformation was performed prior to analysis, and the estimated coefficients were back-transformed for interpretation. All analyses followed the Intention-to-Treat (ITT) principle, whereby all participants were analyzed according to their originally assigned groups, regardless of adherence or dropout.

To assess the robustness of the findings to missing data assumptions, sensitivity analyses were performed using multiple imputation with 10 imputed datasets. The results were pooled using Rubin’s rules in R and compared with those from the primary GEE analyses based on the original data.

A two-tailed p-value of < 0.05 was considered statistically significant.

### 2.9 Ethical Consideration

The research was approved by the Medical Ethics Committee of Zaozhuang Central Hospital of Shandong Guoxin Yiyang Group on 18 October 2024, with approval number 2024−015.

Written informed consent was obtained from all participants prior to enrollment. Participants were fully informed about the purpose, procedures, potential benefits and risks of the study. They were also informed of their right to withdraw from the study at any time without any consequences.

## 3. Results

### 3.1 Participants flow

As illustrated in Fig 1, a total number of 317 patients were screened during recruitment. Of these, 134 eligible individuals provided written informed consent and were enrolled. 134 participants were randomly assigned to the intervention group (n = 67) and the control group (n = 67). Participants were aged 39–74 years, had DKD risk scores between 16 and 29.5, and had lived with diabetes for 1–27 years.

**Fig 1:**
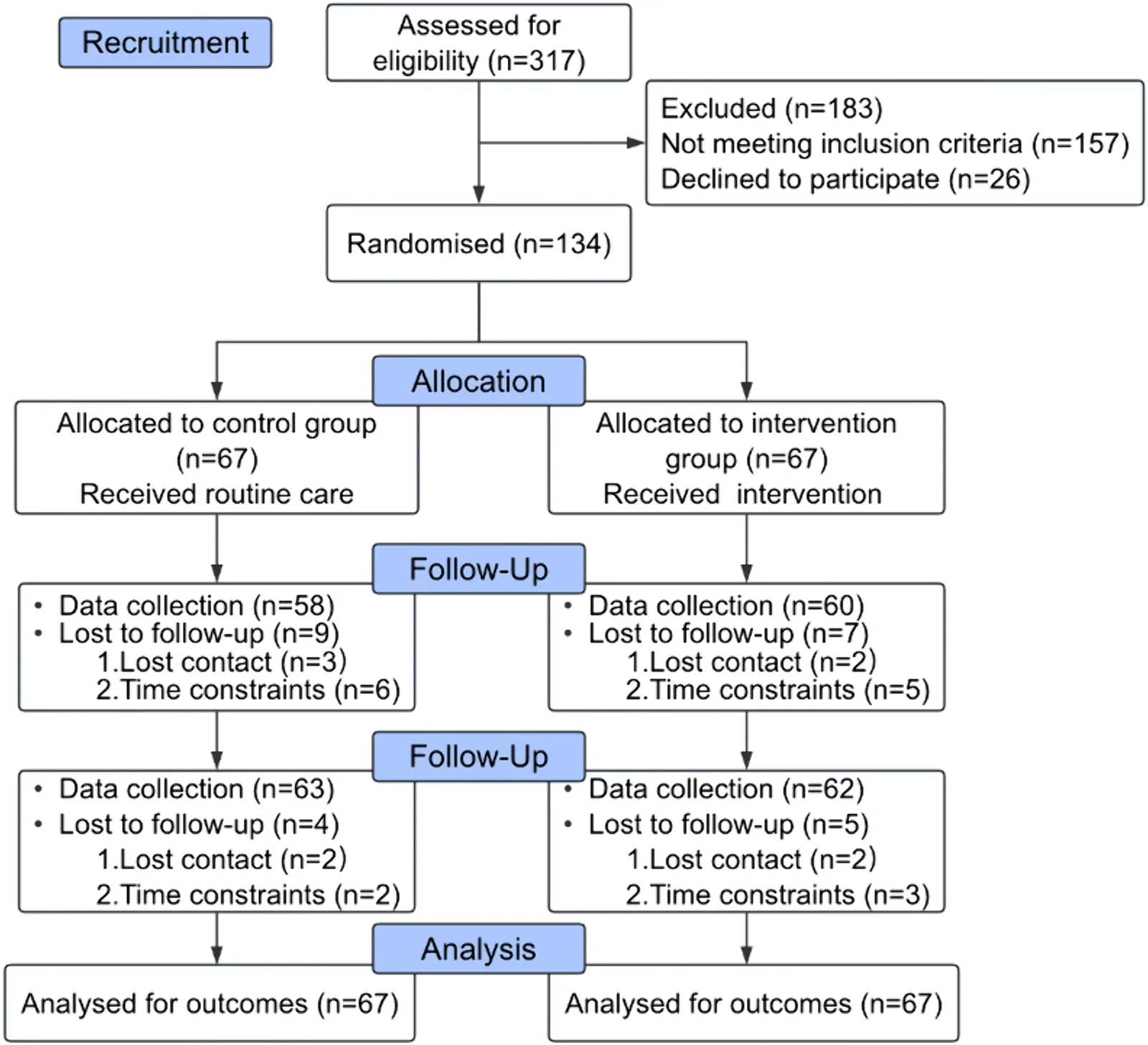
CONSORT flow diagram.

Participants were enrolled from December 2024 to February 2025, and all follow-up assessments were completed by August 2025. All participants received the intervention. During the follow-up visits, some participants did not complete the data collection due to time constraints or loss of contact, these participants were considered lost to follow-up. At the first follow-up, 7 participants in the intervention group and 9 in the control group were lost (attrition: 11.94%). At the second follow-up, 5 intervention participants and 4 control participants were lost (attrition: 6.71%). According to the Intention-to-Treat (ITT) principle, all randomized participants were included in the final analysis.

### 3.2 Baseline Comparison of Lost and Retained Participants

To assess whether participant loss affected study validity, baseline characteristics were compared between those who lost-to-follow-up and retained. As shown in table 2, no significant differences were found in sociodemographic or clinical indicators between the two groups (p > 0.05). This suggests that dropout was random and not associated with baseline characteristics, supporting the assumption of missing-at-random data.

**Table 2:** Baseline comparison between lost-to-follow-up and retained participants.

Variables	Retained (N = 113)	Lost-to-follow-up (N = 21)	statistic value	p
Age (years) ^a^	56.68 ± 8.24	59.19 ± 9.32	−1.256	0.211
Sex^c^				
Male	77(68.1%)	14(66.7%)	0.018	0.894
Female	36(31.9%)	7(33.3%)		
DKD risk score^b^	17.5 (16, 20)	17.5 (16.75, 22.25)	−1.083	0.279
Duration of diabetes^b^	8 (4, 14)	8 (1, 17)	−0.519	0.604
Marital status^c^				
Currently married	103(91.20%)	17(81.0%)	fisher	0.234
Not currently married	10(8.8%)	4(19.0%)		
Education level ^c^				
Primary school or below	12(10.6%)	2(9.5%)	1.238^a^	0.539
Secondary school	61(54.0%)	14(66.7%)		
Undergraduate or above	40(35.4%)	5(23.8%)		
Monthly family income^c^				
3000 RMB or below	12(10.6%)	3(14.3%)	1.680^a^	0.432
3001 to 5000 RMB	46(40.7%)	11(52.4%)		
Above 5001 RMB	55(48.7%)	7(33.3%)		
Residential status^c^				
Living alone	9(8.0%)	3(14.3%)	fisher	0.401
Not living alone	104(92.0%)	18(85.7%)		
Family history of diabetes^c^				
Yes	76(67.3%)	17(81.0%)	1.564^a^	0.211
No	37(32.7%)	4(19.0%)		
History of hypertension^c^				
Yes	54(47.8%)	9(42.9%)	.173^a^	0.678
No	59(52.2%)	12(57.1%)		
Treatment method^c^				
Oral medication	78(69.0%)	17(81.0%)	1.221^a^	0.269
Insulin therapy	35(31.0%)	4(19.0%)		
Frequency of blood glucose monitoring^c^				
Regular monitoring	34(30.1%)	3(14.3%)	2.213^a^	0.137
Irregular monitoring	79(69.9%)	18(85.7%)		
Work intensity^c^				
Light	37(32.7%)	7(33.3%)	.386^a^	0.824
Moderate	50(44.2%)	8(38.1%)		
Heavy	26(23.0%)	6(28.6%)		
HbA1c^b^	8.7 (7.3, 10.8)	9.9 (8.3, 11.7)	−1.843	0.065
eGFR ^a^	101.53 ± 10.14	100.66 ± 11.64	0.353	0.725
UACR^b^	15.84 (6.55, 24.4)	19.75 (13.31, 24.73)	−1.301	0.193
SBP ^a^	134.88 ± 18.15	128.95 ± 17.42	1.384	0.169
LDL-C ^a^	2.62 ± 0.93	2.84 ± 0.79	−1.052	0.295
BMI ^a^	25.66 ± 3.46	24.86 ± 3.57	0.963	0.337
WC^b^	90 (80, 96)	84 (80, 92.5)	−0.549	0.583

Note: ^a^:Continuous variables with normal distribution are presented as Mean ± SD, and the independent samples t-test is used for between-group comparison, with t value as the statistic value. ^b^: Continuous variables with non-normal distribution are presented as Median (P25, P75), and the Mann–Whitney U test is used for between-group comparison, with Z value as the statistic value. ^c^: Categorical variables are presented as frequency (percentage), and the Pearson Chi-Square test or Fisher is used for between-group comparison, with χ² value as the statistic value. HbA1c, hemoglobin A1c (%); UACR, urinary albumin-to-creatinine ratio (mg/g); eGFR, estimated glomerular filtration rate (mL/min/1.73 m²); SBP, systolic blood pressure (mmHg); LDL-C, low-density lipoprotein cholesterol (mmol/L); BMI, body mass index (kg/m²); WC, waist circumference (cm).

### 3.3 Baseline Comparison between the Intervention and Control Groups

To confirm the comparability of the intervention and control groups, baseline characteristics comparison of the two groups is shown in Table 3. No significant differences were observed in any sociodemographic or clinical indicators (p > 0.05), indicating that randomization effectively achieved baseline equivalence between the groups.

**Table 3:** Comparison of sociodemographic and clinical indicators between groups.

Variables	Group		statistic value	P
**Intervention group (n = 67)**	**Control group (n = 67)**
Age (years) ^a^	56.25 ± 8.63	57.90 ± 8.21	−1.129	0.261
Sex^c^			.308	0.579
Male	47(70.1%)	44(65.7%)		
Female	20(29.9%)	23(34.3%)		
DKD risk score^b^	17(16.5 ~ 20)	18(16.5 ~ 21)	−1.036	0.300
Duration of diabetes^b^	7(3 ~ 10)	9(4 ~ 16)	−1.643	0.100
Marital status^c^			2.871	0.090
Currently married	63(94.0%)	57(85.1%)		
Not currently married	4(6.0%)	10(14.9%)		
Education level ^c^			2.352	0.309
Primary school or below	5(7.5%)	9(13.4%)		
Secondary school	36(53.7%)	39(58.2%)		
Undergraduate or above	26(38.8%)	19(28.4%)		
Monthly family income^c^			3.073	0.215
3000 RMB or below	6(9.0%)	9(13.4%)		
3001 to 5000 RMB	25(37.3%)	32(47.8%)		
Above 5001 RMB	36(53.7%)	26(38.8%)		
Residential status^c^			.366	0.545
Living alone	5(7.5%)	7(10.4%)		
Not living alone	62(92.5%)	60(89.6%)		
Family history of diabetes^c^			.879	0.349
Yes	49(73.1%)	44(65.7%)		
No	18(26.9%)	23(34.3%)		
History of hypertension^c^			1.468	0.226
Yes	28(41.8%)	35(52.2%)		
No	39(58.2%)	32(47.8%)		
Treatment method^c^			.036	0.849
Oral medication	48(71.6%)	47(70.1%)		
Insulin therapy	19(28.4%)	20(29.9%)		
Frequency of blood glucose monitoring^c^			1.829	0.176
Regular monitoring	22(32.8%)	15(22.4%)		
Irregular monitoring	45(67.2%)	52(77.6%)		
Work intensity^c^			1.558	0.459
Light	20(29.9%)	24(35.8%)		
Moderate	28(41.8%)	30(44.8%)		
Heavy	19(28.4%)	13(19.4%)		
HbA1c^b^	8.6(7.4 ~ 10.8)	9.3(7.5 ~ 10.8)	−0.725	0.468
eGFR ^a^	103.02 ± 11.42	99.78 ± 8.94	1.828	0.070
UACR^b^	19.58(9.5 ~ 25.34)	15.47(7.71 ~ 23.47)	−1.144	0.253
SBP ^a^	134.34 ± 16.25	133.57 ± 19.90	0.247	0.805
LDL-C ^a^	2.53 ± 0.93	2.77 ± 0.89	−1.566	0.120
BMI ^a^	25.71 ± 3.71	25.36 ± 3.25	0.585	0.560
WC ^a^	89.49 ± 11.88	87.75 ± 11.19	0.876	0.383

Note: ^a^:Continuous variables with normal distribution are presented as Mean ± SD, and the independent samples t-test is used for between-group comparison, with t value as the statistic value. ^b^: Continuous variables with non-normal distribution are presented as Median (P25, P75), and the Mann–Whitney U test is used for between-group comparison, with Z value as the statistic value. ^c^: Categorical variables are presented as frequency (percentage), and the Pearson Chi-Square test is used for between-group comparison, with χ² value as the statistic value. HbA1c, hemoglobin A1c (%); UACR, urinary albumin-to-creatinine ratio (mg/g); eGFR, estimated glomerular filtration rate (mL/min/1.73 m²); SBP, systolic blood pressure (mmHg); LDL-C, low-density lipoprotein cholesterol (mmol/L); BMI, body mass index (kg/m²); WC, waist circumference (cm).

### 3.4 Effects of the Intervention on clinical outcomes between the Two Groups

A significant group-by-time interaction was observed for HbA1c (Wald χ² = 9.693, p = 0.008), indicating that the pattern of change over time differed between the two groups. Further comparisons of estimated marginal means between groups over time revealed that HbA1c level significantly decreased in the intervention group (p = 0.014), whereas no significant change was observed in the control group (p = 0.427). Furthermore, the differences between intervention group and control group were also significant at T1 (p = 0.004) and T2 (p < 0.001). (As shown in table 4)

**Table 4:** Comparison of clinical outcomes between groups over time.

OUTCOMES	IG	CG	LS MD (95% CI)	Wald χ²	P
**HbA1c**					
**T0**	8.80 (8.24–9.40)	9.21 (8.61–9.84)	0.96 (0.88–1.04)	1.056	0.304
**T1**	8.16 (7.64–8.71)	9.33 (8.68–10.01)	0.87 (0.80–0.96)	8.508	0.004
**T2**	7.97 (7.49–8.47)*	9.40 (8.75–10.08)	0.85 (0.78–0.92)	13.813	<0.001
**Wald χ²**	8.606	1.704			
**P**	0.014	0.427			
**UACR**					
**T0**	12.34 (9.99–15.26)	11.23 (9.24–13.63)	1.10 (0.85–1.43)	0.503	0.478
**T1**	8.92 (7.19–11.05)*	10.90 (8.95–13.30)*	0.82 (0.63–1.07)	2.179	0.140
**T2**	8.97 (7.37–10.92)*	11.24 (9.23–13.66)^#^	0.80 (0.62–1.03)	2.931	0.087
**Wald χ²**	210.974	19.487			
**P**	<0.001	<0.001			
**eGFR**					
**T0**	101.20 ± 1.69	99.12 ± 1.22	2.08(−1.37 ~ 5.53)	1.392	0.238
**T1**	100.41 ± 1.67	98.24 ± 1.24	2.16(−1.39 ~ 5.71)	1.428	0.232
**T2**	100.24 ± 1.66*	96.53 ± 1.21*^#^	3.70(0.27 ~ 7.14)	4.479	0.034
**Wald χ²**	13.212	125.734			
**P**	0.001	<0.001			
**SBP**					
**T0**	138.02 ± 2.42	135.07 ± 2.69	2.95(−2.26 ~ 8.16)	1.232	0.267
**T1**	130.78 ± 2.36*	135.41 ± 2.78	−4.62(−9.87 ~ 0.62)	2.983	0.084
**T2**	130.54 ± 2.24*	136.08 ± 2.62	−5.55(−10.49 ~ −0.61)	4.847	0.028
**Wald χ²**	2063.443	3.928			
**P**	<0.001	0.140			
**LDL- C**					
**T0**	2.39 ± 0.12	2.66 ± 0.12	−0.269(−0.554 ~ 0.0164)	3.412	0.065
**T1**	2.09 ± 0.12*	2.54 ± 0.12	−0.4473(−0.7262 ~ −0.1684)	9.879	0.002
**T2**	2.07 ± 0.12*	2.62 ± 0.11	−0.5495(−0.8192 ~ −0.2797)	15.935	<0.001
**Wald χ²**	32.695	4.961			
**P**	<0.001	0.084			
**BMI**					
**T0**	25.75 ± 0.52	25.24 ± 0.42	0.5192(−0.5583 ~ 1.5968)	0.892	0.345
**T1**	25.45 ± 0.52*	25.34 ± 0.43	0.1065(−0.9635 ~ 1.1766)	0.038	0.845
**T2**	25.11 ± 0.53*^#^	24.96 ± 0.43*	0.1489(−0.9416 ~ 1.2393)	0.072	0.789
**Wald χ²**	436.190	495.953			
**P**	<0.001	<0.001			
**WC**					
**T0**	88.81 ± 1.58	86.72 ± 1.25	2.09(−1.31 ~ 5.50)	1.449	0.229
**T1**	87.64 ± 1.60*	85.98 ± 1.39	1.66(−1.97 ~ 5.30)	0.804	0.370
**T2**	86.56 ± 1.57*^#^	84.84 ± 1.40*^#^	1.71(−1.94 ~ 5.37)	0.844	0.358
**Wald χ²**	67.501	50.198			
**P**	<0.001	<0.001			

Note: 1. Values are presented as least-square means estimated from the GEE model. For normally distributed variables, results are expressed as mean ± standard error (SE), whereas for log-transformed variables (HbA1c and UACR), results are presented as back-transformed means with 95% confidence intervals (CI). 2. LS MD represents the adjusted between-group difference (Intervention − Control) at each time point. For normally distributed variables, LS MD is expressed as mean difference (95% CI), whereas for log-transformed variables, LS MD is expressed as the ratio (95% CI) after back-transformation. 3. Pairwise comparisons of estimated marginal means were performed. * Indicates a significant difference compared with baseline (T0), and # indicates a significant difference compared with 3 months (T1). 4. All models were adjusted for education level, family history of diabetes, diabetes duration, and DKD risk score. GEE models were specified with a normal distribution and identity link function, and robust standard errors were applied. 5. IG indicates intervention group; CG indicates control group.

A significant group-by-time interaction was observed for UACR (Wald χ² = 151.087, p < 0.001), indicating that the pattern of change over time differed between the intervention and control groups. Further comparisons of estimated marginal means showed that UACR levels changed significantly over time in both groups, with a greater reduction observed in the intervention group compared with the control group. However, no significant between-group differences were observed at T1 (p = 0.140) or T2 (p = 0.087). These findings suggest that although the trajectories of change differed between groups, the differences at individual time points did not reach statistical significance. (As shown in table 4)

A significant group-by-time interaction was observed for eGFR (Wald χ² = 29.047, p < 0.001), indicating that the pattern of change over time differed between the intervention and control groups. Further comparisons of estimated marginal means showed that eGFR significantly decreased from T0 to T2 in both groups (intervention group: p = 0.001; control group: p < 0.001). However, the magnitude of decline was smaller in the intervention group (from 101.20 to 100.24) compared with the control group (from 99.12 to 96.53), suggesting a slower deterioration of renal function. In addition, a significant between-group difference was observed at T2 (p = 0.034). (As shown in table 4)

A significant group-by-time interaction was observed for SBP (Wald χ² = 351.386, p < 0.001), indicating that the pattern of change over time differed between the intervention and control groups, with greater improvement observed in the intervention group. Further comparisons of estimated marginal means showed that SBP significantly decreased from T0 to T1 and T2 in the intervention group (p < 0.001), whereas no significant change was observed in the control group (p = 0.140). In addition, a significant between-group difference was observed at T2 (p = 0.028). (As shown in table 4)

A significant group-by-time interaction was observed for LDL-C (Wald χ² = 12.140, p = 0.002), indicating that the pattern of change over time differed between the intervention and control groups, with greater improvement observed in the intervention group. Further comparisons of estimated marginal means of LDL- C levels revealed that LDL- C levels significantly decreased from T0 to T1 and T2 in the intervention group (p < 0.001), whereas no significant change was observed in the control group (p = 0.084). Moreover, the differences between intervention group and control group were also significant at T1 (p = 0.002) and T2 (p < 0.001). (As shown in table 4)

A significant group-by-time interaction was observed for BMI (Wald χ² = 25.029, p < 0.001), indicating that the pattern of change over time differed between the intervention and control groups. Further comparisons of estimated marginal means showed that both groups experienced significant reductions in BMI over time (p < 0.001). However, no significant between-group differences were observed at T1 (p = 0.845) or T2 (p = 0.789), suggesting that the intervention did not produce a greater reduction in BMI compared with usual care. (As shown in table 4)

The group-by-time interaction for WC was not statistically significant (Wald χ² = 0.702, p = 0.704), indicating that the pattern of change over time did not differ between the intervention and control groups. Therefore, the main effects were interpreted. A significant main effect of time was observed (Wald χ² = 110.585, p < 0.001), indicating that WC levels decreased over time across both groups. However, the main effect of group was not significant (Wald χ² = 1.024, p = 0.312), suggesting no overall difference between the intervention and control groups. Further comparisons of estimated marginal means of WC revealed that there were no significant between-group differences at T1 (p = 0.370) or T2 (p = 0.358). These findings indicate that although WC improved over time, the intervention did not produce a greater reduction compared with usual care.

### 3.5 Sensitivity Analysis

Sensitivity analyses were conducted using multiple imputation with 10 imputed datasets. The results of the sensitivity analyses were consistent with those obtained from the primary GEE analyses based on the original data. Specifically, significant group-by-time interactions remained for HbA1c, UACR, eGFR, LDL-C, SBP, and BMI, whereas no significant interaction was observed for WC. Similarly, the patterns of between-group differences at individual time points were largely unchanged; at T2, significant between-group differences were observed for HbA1c, eGFR, LDL-C, and SBP, but not for UACR, BMI, or WC, consistent with the primary analysis. These findings suggest that the study results were robust to the handling of missing data. Detailed results are presented in S5.

## 4. Discussion

### 4.1 Effects of self-management intervention on clinical outcomes

The GEE analysis of HbA1c revealed a significant group effect and a significant group-by-time interaction, indicating that the self-management intervention achieved better glycemic control over time. Specifically, HbA1c levels in the intervention group decreased significantly from 8.80% at T0 to 7.97% at T2 (p = 0.014), while the control group showed a slight increase from 9.21% to 9.40% (p = 0.427). At T2, HbA1c in the intervention group was approximately 15% lower than in the control group (ratio = 0.85, 95% CI: 0.78–0.92), indicating a substantial intervention effect. The observed reduction in HbA1c in the intervention group is clinically meaningful, suggesting that the self-management program effectively improved glycemic regulation. This intervention emphasized the importance of glycemic control in preventing DKD progression and highlighted participants’ susceptibility to DKD. By increasing their awareness of personal risk, the intervention promoted behavioral change and strengthened self-management adherence, which may have contributed to the observed improvements in metabolic outcomes.

The findings are consistent with previous studies reporting the effectiveness of diabetes self-management education in improving glycemic control. For instance, a randomized controlled trial among Indian patients demonstrated a significant HbA1c reduction from 9.0% to 7.7% after a 3-month diabetes self-management education program (mean difference: –1.3%, SD: 0.4) [31]. This is consistent with a recent meta-analysis showing a pooled standardized mean difference of –0.604 (95% CI: –0.854 to –0.353, *p* < 0.001) across multiple self-management intervention studies, suggesting a clinically meaningful improvement in glycemic outcomes [32]. However, despite the significant improvement, the mean HbA1c in the intervention group remained above the optimal target (<7.0%), indicating that while the intervention was effective, additional or longer-term strategies may be necessary to achieve and sustain optimal glycemic targets in this population. Evidence has confirmed that HbA1c levels above 7.2% are associated with an increased risk of microvascular complications, highlighting the importance of achieving tighter glycemic control [33]. Poor glucose control also contributed to the risk of chronic kidney disease and associated with more rapid renal function decline [34,35]. Factors such as disease chronicity, medication complexity, and psychosocial barriers might have limited the extent of HbA1c reduction.

The present study found that UACR tended to decrease in the intervention group following the self-management intervention, whereas minimal change was observed in the control group. The significant time effect and group-by-time interaction indicate that UACR levels changed differently between the two groups over time. Specifically, the intervention group showed a reduction trend in UACR from 12.34 mg/g at baseline to 8.97 mg/g at 6 months (p < 0.001), whereas the control group also exhibited a significant but less pronounced change over time. Moreover, no significant between-group differences were observed at both T1 and T2, suggesting that the intervention may have influenced the trajectory of UACR over time rather than producing distinct differences at individual time points.

These findings are consistent with previous research, which reported that self-management interventions significantly reduced UACR at 3 years, although the effect was not sustained in the longer term (4 years), suggesting that improvements in renal biomarkers may weaken over time, potentially due to reduced adherence or diminishing behavioral influence [36]. The intervention emphasized practical skills in diet control, blood glucose monitoring, and medication adherence, which may have improved metabolic stability and alleviated renal stress. HbA1c improvement observed in the intervention group further supports this interpretation, as better glycemic control is strongly associated with a reduction in albuminuria and slower kidney damage progression.

In this study, both the intervention and control groups showed a statistically significant decline in eGFR, which is physiologically expected among patients with T2DM. However, the decline was notably slower in the intervention group. Specifically, eGFR decreased slightly from 101.20 to 100.24 mL/min/1.73 m² in the intervention group, whereas the control group experienced a larger reduction from 99.12 to 96.53 mL/min/1.73 m², with a significant between-group difference at T2, suggesting that the self-management intervention helped attenuate renal function decline. These findings align with a long-term study, in which structured diabetes self-management education among patients with microalbuminuria resulted in better preservation of eGFR over four years compared with usual care [36].

The slower decline in eGFR in the intervention group may reflects the benefits of improved metabolic control, better medication adherence, and healthier lifestyle behaviors. Enhanced glycemic and blood pressure management can reduce intraglomerular pressure and hyperfiltration, thereby slowing renal damage. Although a slight decrease in eGFR was observed, the change was clinically minimal and within the expected range for stable renal function. These findings suggest that the intervention may help maintain renal function and delay DKD progression. Continuous reinforcement and long-term follow-up are recommended to sustain these effects.

In this study, a significant group-by-time interaction effect in SBP was found, with the between-group difference at T2 was also significant, indicating that the self-management intervention effectively improved blood pressure control over time compared with usual care. These findings are consistent with previous evidence showing that self-management or lifestyle interventions can significantly reduce blood pressure in patients with T2DM [37]. Likewise, a meta-analysis reported that diabetes self-management education significantly reduces SBP, with the greatest effects observed in interventions emphasizing diet, exercise, and medication adherence [38]. The improvement in SBP in the present study may be attributed to enhanced diet, physical activity, and medication adherence promoted through the education program. The observed SBP reduction is clinically meaningful, given that elevated blood pressure is a key determinant of DKD progression [14,34]. Reduction in SBP is considered a key strategy in DKD management, as elevated SBP contributes to glomerular hyperfiltration, albuminuria, and progressive renal function decline [39,40]. Therefore, the observed SBP improvement in the intervention group aligns with the concurrent slower decline in eGFR, suggesting that the intervention may indirectly contribute to renal protection by maintaining optimal blood pressure levels.

In the present study, LDL-C levels demonstrated significant group-by-time interaction effects, indicating that the self-management intervention led to a greater reduction in LDL-C over time compared with usual care. Specifically, LDL-C levels in the intervention group decreased significantly from baseline (2.39 ± 0.12 mmol/L) to T1 (2.09 ± 0.12 mmol/L) and T2 (2.07 ± 0.12 mmol/L) (p < 0.001), whereas no significant change was observed in the control group (p = 0.084). These findings are consistent with previous studies, which reported that structured education programs effectively improve both glycemic and lipid profiles in individuals with T2DM [41]. Given that LDL-C is a known risk factor for DKD, intensive LDL-C reduction is associated with a lower risk of developing DKD in T2DM patients [42]. In the present study, participants in the intervention group received education emphasizing dietary modification, physical activity, and medication adherence, which likely contributed to the observed LDL-C reduction and may have implications for DKD risk management.

Although the interaction effect was significant, there were no differences in BMI and WC between the intervention and control groups at T1 or T2, suggesting that the intervention did not result in statistically significant between-group differences in these indicators. This finding is consistent with previous studies, which have reported that lifestyle or self-management interventions in patients with diabetes often do not lead to significant differences in BMI between intervention and control groups [43,44]. Similarly, a systematic review found that smartphone-based self-management interventions did not result in statistically significant improvements in BMI among patients with T2DM [45]. One possible explanation for the absence of significant between-group differences is that this intervention was not specifically designed for weight reduction. The dietary and exercise topics focused on fostering long-term healthy eating habits and regular exercise rather than achieving short-term weight loss. Therefore, although participants in the intervention group have improved their dietary and exercise behaviors, the magnitude of these changes was insufficient to produce significant between-group differences within six months. Nevertheless, the within-group reductions in BMI and WC remain clinically meaningful, especially given the high baseline WC among participants and its strong association with metabolic and cardiovascular risk [46,47]. To achieve more pronounced improvements in body weight or central obesity in the future, more stringent calorie-restricted dietary plans and structured exercise programs may be necessary.

### 4.2 Limitations of Study

This study lasted 24 weeks, including a 12-week intervention and a subsequent 12-week follow-up. Due to time constraints, long-term follow-up was not conducted, and the follow-up period may therefore be insufficient to assess the impact of the intervention on hard endpoints such as end-stage renal disease or other long-term renal outcomes. The findings indicate that the short-term intervention effectively improved blood glucose and lipid profiles, providing a foundation for achieving long-term benefits.

### 4.3 Implications for Clinical Practice

From the perspective of clinical practice, incorporating DKD risk assessment into the routine care of patients with T2DM facilitates the early identification of individuals at high risk. Delivering targeted self-management education to these patients can effectively improve their awareness of DKD risk and strengthen their self-management behaviors. As the intervention was delivered through standardized video-based education, it can be readily implemented in both tertiary hospitals and primary care settings, supporting the wider dissemination of early DKD prevention programmes.

## 5. Conclusion

This is the first study to evaluate the effectiveness of a theory-based self-management intervention among patients with T2DM who are at high risk of DKD. The intervention significantly improved HbA1c, eGFR, SBP, and LDL-C and was associated with favorable changes in UACR over time. These findings support the integration of structured self-management into routine diabetes care as a strategy for early DKD prevention, while longer-term studies are warranted to confirm its sustained effects on renal outcomes.

**What is already known**

- The rising prevalence of T2DM has led to a significant increase in the number of individuals affected by DKD.
- DKD exerts a substantial negative impact on patients due to its progressive nature and associated complications.
- Currently, self-management interventions for DKD patients primarily focus on the microalbuminuria and dialysis stages.

**What this paper adds**

- This study is the first to target patients with T2DM at high risk of developing DKD, using a risk prediction model to identify high risk individuals and incorporate personalized risk awareness into self-management interventions.
- Intervention content was systematically developed based on clinical guidelines and explicitly mapped to the constructs of the Health Belief Model, ensuring theoretical alignment and practical relevance across diet, exercise, medication, and self-monitoring.
- The study is the first to assess chronic kidney disease risk perception in an interventional context using a validated, theoretically aligned instrument, demonstrating that aligning high-risk populations, theoretical frameworks, and risk perception measures can enhance the effectiveness of behavioral interventions.

## Supporting information

S1 Appendix Trial Protocol. (DOCX)

S2 Appendix Technical Review Application. (DOCX)

S3 Appendix Ethics Approval – Original version and English-translated version. (DOCX)

S4 Appendix CONSORT checklist. (DOCX)

S1 Table Consistency of results between generalized estimating equations analyses based on original (non-imputed) data and multiple imputation analyses, including group-by-time interactions and between-group differences at each time point. (DOCX)

S1 Fig Changes in outcomes over time in the intervention and control groups based on GEE models. (DOCX)

S5 Appendix Inclusivity in global research. (DOCX)

S1 Dataset De-identified participant-level dataset used for the analyses in this study. (XLSX)
