# Supplementary material for: Effectiveness of a self-management intervention on clinical outcomes among patients with type 2 diabetes at high risk for Diabetic Kidney Disease: A Randomised Controlled Trial
Source: PLoS One. 2026 Oct 12;21(10):e0360475. doi: 10.1371/journal.pone.0360475 (PMC13657325; doi:10.1371/journal.pone.0360475)
Supplement: Supplementary file 1 [file pone.0360475.s009.pdf]

**Effectiveness of a Self-Management Intervention on Clinical Outcomes Among Patients With Type 2 Diabetes at High Risk of Diabetic Kidney Disease: A Randomized Controlled *Trial***

## Reviewer Report

**Research Title:** The research title, *"Effectiveness of a Self-Management Intervention on Clinical Outcomes Among Patients With Type 2 Diabetes at High Risk of Diabetic Kidney Disease: A Randomized Controlled Trial,"* is clear and accurately reflects the study's focus, design, and population. It highlights both the intervention and the clinical outcomes of interest, and the inclusion of "randomized controlled trial" signals methodological rigour. The title is informative but could be slightly streamlined for readability, for example by condensing "Patients With Type 2 Diabetes at High Risk of Diabetic Kidney Disease" to "High-Risk Type 2 Diabetes Patients."

**Manuscript Assessment:** Overall, the manuscript addresses an important clinical issue, as diabetic kidney disease (DKD) is a major complication of type 2 diabetes and contributes to significant morbidity, mortality, and healthcare costs. By focusing on early-stage interventions targeting modifiable risk factors, the study provides evidence on preventive strategies that could reduce progression to DKD. The randomised controlled trial design adds robustness, and the results have potential implications for clinical practice, particularly in guiding patient self-management programmes and informing healthcare policy in diabetes care.

**Abstract:** The abstract effectively summarises the study but could be improved. First, the background could briefly emphasise the gap in knowledge regarding early-stage DKD prevention. Second, the objectives should be clearly framed as a statement of research aim. Third, the methods could specify the randomisation procedure and provide brief information about ethical approvals. Fourth, the results section should clearly report effect sizes and statistical significance for each outcome. Fifth, the conclusions could more explicitly link the findings to practical recommendations for clinical care and patient self-management.

**Introduction:** The introduction would benefit from additional context on the global and regional burden of DKD, the pathophysiological mechanisms linking T2DM to kidney disease, and the current state of evidence for self-management interventions. The rationale for selecting high-risk T2DM patients should be elaborated, highlighting why early intervention is particularly important. A clear statement of the research gap and explicit study objectives would strengthen the introduction.

**Methods:** The methodology section is generally well described but requires further detail for reproducibility. Clarification is needed regarding inclusion and exclusion criteria, recruitment procedures, and consent/ethical approval. The randomisation process, allocation concealment, and blinding should be described. Details on the self-management intervention, its components, frequency, delivery method, and adherence monitoring should be expanded. Statistical analyses, including handling of missing data and assumptions for generalised estimating equations, should be clearly stated.

**Results:** The results section presents outcomes effectively but could be improved in clarity and organisation. Tables should clearly display baseline characteristics, attrition rates, and outcome measures at each time point, with effect sizes and confidence intervals. Highlighting clinically

meaningful improvements alongside statistical significance would enhance interpretation. Any secondary analyses or subgroup findings could be briefly summarised. Visualisations, such as line graphs for longitudinal outcomes, could improve readability.

**Discussion:** The discussion section should interpret findings in the context of existing literature, emphasising both the novelty and consistency of the results. Potential mechanisms by which the intervention improved clinical outcomes should be elaborated. Limitations, including attrition, generalisability, and intervention fidelity, should be clearly acknowledged. The discussion should also outline how the findings can inform clinical practice, policy development, and patient education in diabetes care.

**Study implications:** The study implications section could be strengthened by specifying how healthcare providers might implement self-management interventions, including integrating lifestyle education, monitoring protocols, and patient engagement strategies. Consideration of cost-effectiveness and feasibility in routine clinical settings would enhance relevance. Highlighting the potential to delay DKD progression and reduce healthcare burden would reinforce the practical impact.

**Future research directions:** Future research directions could include evaluating long-term sustainability of clinical improvements, exploring intervention effectiveness in different healthcare settings or populations, comparing different types of self-management interventions, and assessing patient adherence and engagement strategies. Research on digital or remote self-management tools could also be suggested.

**Referencing:** The reference section should be reviewed for completeness, currency, and adherence to the journal’s style. Inclusion of recent literature on DKD prevention, self-management interventions, and T2DM outcomes would strengthen the scientific context. Cross-checking citations with the reference list is recommended.

**Proofreading and editing:** Proofreading and editing are recommended to improve clarity, consistency, and readability. Grammar, sentence structure, and terminology should be standardised. Tables and figures should have consistent formatting and appropriate labels. Minor revisions for conciseness and logical flow would enhance overall presentation and manuscript quality.
