# Supplementary material for: Effectiveness of a self-management intervention on clinical outcomes among patients with type 2 diabetes at high risk for Diabetic Kidney Disease: A Randomised Controlled Trial
Source: PLoS One. 2026 Oct 12;21(10):e0360475. doi: 10.1371/journal.pone.0360475 (PMC13657325; doi:10.1371/journal.pone.0360475)
Supplement: Supplementary file 2 [file pone.0360475.s011.pdf]

Effectiveness of a Self-Management Intervention on Clinical Outcomes Among Patients with Type 2 Diabetes at High Risk of Diabetic Kidney Disease: A Randomized Controlled Trial

Peer Reviewer Report

1. Overall Assessment of the Research

This manuscript addresses an important clinical and public health issue by evaluating whether a structured self-management intervention can improve clinical outcomes among patients with type 2 diabetes mellitus who are at elevated risk of diabetic kidney disease. The study is highly relevant because diabetic kidney disease remains one of the most common and costly complications of diabetes globally, and preventive strategies targeted at high-risk individuals are urgently needed. The randomised controlled trial design strengthens the internal validity of the study and improves the reliability of causal inference. The findings contribute important evidence supporting early-stage behavioral and clinical management interventions before overt diabetic kidney disease develops. The manuscript is well structured and clinically meaningful. However, several areas require clarification and deeper analytical interpretation, particularly in the reporting of intervention components, outcome measurement, attrition handling, and interpretation of results. With revision, this manuscript would make a valuable contribution to diabetes care and chronic disease prevention literature.

2. Summary of the Entire Research

This study examined the effectiveness of a structured self-management intervention among adults with type 2 diabetes at high risk of diabetic kidney disease in a tertiary hospital in China. A two-arm randomised controlled trial was conducted with 134 participants assigned equally to intervention and control groups. The intervention group received a 12-week structured self-management programme followed by a 12-week follow-up, while the control group received standard care. Clinical outcomes including HbA1c, urinary albumin-to-creatinine ratio, estimated glomerular filtration rate, systolic blood pressure, LDL cholesterol, body mass index, and waist circumference were assessed at baseline, post-intervention, and follow-up. The intervention improved HbA1c, eGFR, systolic blood pressure, and LDL cholesterol compared with usual care. No significant improvements were observed for urinary albumin-to-creatinine ratio, body mass index, or waist circumference. The findings suggest that structured self-management may reduce modifiable risk factors associated with diabetic kidney disease progression and may contribute to delaying disease onset.

3. Major Comments

Research title

The title is clear, specific, and methodologically precise. It communicates the study population, intervention focus, outcome direction, and study design effectively. It is suitable for journal publication. The only possible refinement would be shortening slightly for readability, for example:

"Effectiveness of a Self-Management Intervention on Clinical Outcomes Among Patients with Type 2 Diabetes at High Risk for Diabetic Kidney Disease: A Randomised Controlled Trial."

Abstract:

1. The abstract should clearly define what "high risk of diabetic kidney disease" means. What diagnostic or clinical criteria were used?
2. The intervention requires clearer description. What specific components were included such as education, counselling, monitoring, dietary modification, or medication adherence support?
3. The attrition numbers need correction and clarification because the reported "539 participants lost to follow-up" appears inconsistent.
4. The magnitude of effect should be reported. Include adjusted estimates, confidence intervals, or effect sizes rather than only stating significant improvement.
5. The conclusion should briefly note the practical implication for diabetes management and whether the intervention could be integrated into routine hospital care.

Introduction:

1. Does the introduction sufficiently describe the global and regional burden of diabetic kidney disease among people with type 2 diabetes?
2. Give us global prevalence of diabetic kidney disease and the issues that led to the low or high prevalence increase in high-income countries and Low-middle-income countries.
3. Can more evidence be added on why early intervention before clinically established diabetic kidney disease is especially important?

4. What is currently known about self-management interventions specifically among high-risk diabetic kidney disease populations?
5. How does this study differ from previous diabetes self-management trials?
6. Why was China selected as the study setting, and what contextual health system factors make this research important?
7. Are theoretical or behavioural frameworks guiding the intervention clearly explained?
8. Is the clinical importance of HbA1c, eGFR, and UACR sufficiently justified?
9. Can stronger justification be provided for targeting modifiable risk factors?
10. Is the research gap clearly articulated and distinct from general diabetes self-management literature?
11. Are the study objectives and hypotheses explicitly stated at the end of the introduction?

Methodology:

1. How was randomisation performed and who generated allocation?
2. Was allocation concealment used?
3. Were participants or assessors blinded?
4. How was sample size calculated and was power sufficient?
5. How was "high risk of diabetic kidney disease" operationalised?
6. What were inclusion and exclusion criteria?
7. What exactly did the intervention contain and how often was it delivered?
8. Was intervention fidelity monitored?
9. How was missing data handled beyond intention-to-treat analysis?
10. Was ethics approval and trial registration clearly reported?

Results and tables:

1. Correct participant flow numbers and ensure consistency.
2. Include a CONSORT flow diagram.
3. Present baseline demographic characteristics clearly in one table.
4. Report adjusted estimates with confidence intervals.

5. Clarify within-group and between-group comparisons.
6. Include exact p values where possible.
7. Present intervention adherence data.
8. Show outcome trends across T0, T1, and T2 clearly.
9. Indicate clinical significance, not only statistical significance.
10. Explain why UACR, BMI, and WC did not improve.
11. Ensure all tables use consistent decimal places and units.
12. Add supplementary sensitivity analysis outputs if journal permits.

Discussion:

1. Begin with the most important findings.
2. Explain why HbA1c improved.
3. Explain why eGFR improved despite short follow-up.
4. Interpret LDL and blood pressure improvements clinically.
5. Discuss why BMI and waist circumference did not improve.
6. Compare findings with prior randomized trials.
7. Discuss intervention feasibility in tertiary hospitals.
8. Consider whether effects may differ in primary care.
9. Address possible selection bias.
10. Discuss attrition implications.
11. Explain how findings support diabetic kidney disease prevention.
12. End with a balanced conclusion.

Strengths and Limitations

Strengths

The randomized controlled design strengthens causal inference. The study targets an understudied but clinically important high-risk population. Multiple objective clinical outcomes were measured. Intention-to-treat and sensitivity analyses improve methodological rigour. The follow-up period strengthens evidence on short-term sustainability.

Limitations

Single-site recruitment may reduce generalisability. Short follow-up limits long-term interpretation. Attrition requires clearer explanation. Lack of blinding may introduce bias. Behavioural adherence measures were not fully reported.

Study implications:

1. How can this intervention be integrated into routine diabetes clinics in China?
2. What are implications for preventing progression to diabetic kidney disease?
3. Could this intervention be adapted for broader community-based diabetes care?

Future research directions:

1. Can effects be sustained beyond six months?
2. Would similar outcomes be observed in multicenter studies?
3. Which intervention components are most effective?
4. What is the cost-effectiveness of implementation?

Reference section:

1. Are all recent diabetic kidney disease prevention trials cited?
2. Are references consistently formatted?
3. Are foundational diabetes self-management guidelines included?

Proofreading and editing recommendations

1. Correct typographical and numerical inconsistencies.
2. Improve consistency of terminology across manuscript.
3. Tighten sentence structure for clarity and flow.

Recommended articles for strengthening references

1. Zimbudzi, E., Lo, C., Misso, M. L., Ranasinha, S., Kerr, P. G., Teede, H. J., & Zoungas, S. (2018). Effectiveness of self-management support interventions for people with comorbid diabetes and chronic kidney disease: A systematic review and meta-analysis. *Systematic Reviews*, *7*, 84. https://doi.org/10.1186/s13643-018-0748-z

2. Waki, K., Nara, M., Enomoto, S. *et al.* Effectiveness of DialBetesPlus, a self-management support system for diabetic kidney disease: Randomized controlled trial. *npj Digit. Med.* **7**, 104 (2024). https://doi.org/10.1038/s41746-024-01114-8

3. Khavere, S., Hadjiconstantinou, M., Miksza, J., Hagan, J., Naderpour, S., Hassen, S. N., Karimi, Z., & Gillies, C. L. (2025). Effectiveness of self-management interventions on Type 2 diabetes among young adults (18–45 years): A systematic review and meta-analysis. *Diabetic Medicine*, *42*(10), e70127. https://doi.org/10.1111/dme.70127

4. Gathu, Catherine W., Shabani, Jacob, Kunyiha, Nancy, & Ratansi, Riaz. (2018). Effect of diabetes self-management education on glycaemic control among type 2 diabetic patients at a family medicine clinic in Kenya: A randomised controlled trial. *African Journal of Primary Health Care & Family Medicine* , *10*(1), 1-9. https://doi.org/10.4102/phcfm.v10i1.1762

5. Edward Zimbudzi, Clement Lo, Sanjeeva Ranasinha, Peter G. Kerr, Timothy Usherwood, Alan Cass, Gregory R. Fulcher, Sophia Zoungas (2017). Self-management in patients with diabetes and chronic kidney disease is associated with incremental benefit in HRQOL. Journal of Diabetes and its Complications, Volume 31, Issue 2. Pages 427-432. ISSN 1056-8727, https://doi.org/10.1016/j.jdiacomp.2016.10.027. (https://www.sciencedirect.com/science/article/pii/S1056872716303920)

Overall, this is a valuable and clinically relevant manuscript. The trial design and focus on early prevention are strong. With clarification of intervention details, correction of participant flow reporting, and deeper interpretation of findings, the manuscript would be substantially strengthened and suitable for journal publication.
