# Supplementary material for: Effectiveness of a self-management intervention on clinical outcomes among patients with type 2 diabetes at high risk for Diabetic Kidney Disease: A Randomised Controlled Trial
Source: PLoS One. 2026 Oct 12;21(10):e0360475. doi: 10.1371/journal.pone.0360475 (PMC13657325; doi:10.1371/journal.pone.0360475)
Supplement: Supplementary file 3 [file pone.0360475.s001.docx]

**Effect of Self-Management Intervention among Patients with Type 2 Diabetes at High Risk of Diabetic Kidney Disease: Protocol for Randomized Controlled Trial**

**(Ethics-approved version)**

**Methods**

1 Study design

The study design is a prospective, two-arm, assessor-blinded, randomized controlled trial. It was registered in the China Clinical Trial Registry on 22 November 2024, with registration number ChiCTR2400092745.

2 Participants

2.1 Inclusion criteria

1) All patients meet the World Health Organization diagnostic criteria for T2DM.
2) Patients at high risk for DKD (DKD risk scores ≥16) (Jiang W et al., 2020).
3) Age 39–75 years old.
4) Patients can use WeChat.
5) Voluntarily join the study and sign the informed consent form.

2.2 Exclusion criteria

1) Patients with DKD.
2) Those who have mental disorders, cognitive impairment, etc. and are unable to cooperate with the researcher.
3) Other secondary glomerular diseases caused by primary renal disease and hypertension, renal arteriosclerosis.
4) There have been recent factors affecting urinary protein such as diabetic ketoacidosis, acute nephritis, and urinary tract infection.

3 Sample size

$$n=\frac{2\sigma^{2}{[Z_{1-\alpha/2}+Z_{1-\beta}]}^{2}}{\left( u_{1}-u_{2} \right)^{2}}$$

Where:

Z_1-α/2_ = Z statistic for level of confidence of 95% = 1.96,

Z_1-β_ = Z statistic for 80% of power = 0.842,

σ is the standard deviation of the outcome variable, $\sigma=\sqrt{\frac{{\sigma_{1}}^{2}+{\sigma_{2}}^{2}}{2}}$

$u_{1}$ , $u_{2}$ are means of intervention group before and after the intervention,

According to Thojampa S (2017), the baseline HbA1c for the intervention group patients was 7.79 ± 1.82. After the 12-week self-management program, the average decreased to 7.06 ± 0.89. The calculation resulted in n≈60, with 60 individuals per group, resulting a total of 120 individuals across two groups. Consideration of 90% response, n ≈134.

Hence, sample size of 134 will be used for.

4 Recruitment and randomization

The recruitment poster is placed at the nurses' station for patient enrollment, allowing all patients to know about the study and express their interest. Potential participants who are interested in the study will be evaluated by a researcher to determine whether they meet the inclusion criteria. Individuals who met the eligibility criteria and agreed to participate provided written informed consent (see Supplementary File 1) before enrollment. Eligible participants were then randomly assigned to the intervention or control group. The enrolled population may be significantly smaller than the screened population; therefore, screening will continue until the target sample size is reached. The recruitment plan began on 2 December 2024, and the recruitment period is expected to last three months.

For randomization, simple randomization is applied. A non-researcher uses an online random number generator (<https://www.randomizer.org/>) to generate 134 unique random integers, which correspond to the enrollment sequence numbers. The enrollment number is labeled on the outside of the kraft paper envelope, with the corresponding random numbers placed inside. A researcher screens patients based on the inclusion and exclusion criteria, introduce the purpose and procedures of the study. Patients who sign the informed consent will receive an envelope corresponding to their enrollment number. Odd random numbers will be assigned to the intervention group, while even numbers will be assigned to the control group.

5 Blinding

This study will be a single-blind RCT. Due to the nature of the intervention, blinding is not feasible for researchers responsible for recruitment and allocation, the intervention implementers, or the data analysts. To minimize bias, outcome assessors and participants will be blinded. They will identify participants using the enrollment number rather than the randomization number when collecting data, which is a feasible approach in this study.

6 Intervention

6.1 Intervention group

The intervention team consists of one endocrinologist, one nursing manager, two diabetes-specialized nurses, and a nursing researcher. One researcher is responsible for participant recruitment and group allocation, two researchers are in charge of data collection, one researcher is responsible for implementing the intervention and statistical analysis, and one researcher assists with statistical analysis and overall study supervision. To ensure the uniformity and effectiveness of the intervention, all members of the intervention team will be trained before the implementation of the intervention. The training covered topics such as patient inclusion criteria, grouping methods, allocation concealment methods, intervention implementation procedures, and considerations for data collection.

**6.2 Intervention Content**

In addition to the control group, participants in the intervention group receive self-management education program (SMEP), which covers five topics: 1) DKD knowledge, 2) diet management, 3) physical activity, 4) medication management, and 5) self-monitoring. The content of SMEP topics is structured based on evidence, referencing related clinical guidelines for diabetes and chronic kidney disease, including "Standards of Care in Diabetes-2024" (American Diabetes Association Professional Practice Committee, 2024), “KDIGO 2022 clinical practice guideline for diabetes management in chronic kidney disease” (Kidney Disease: Improving Global Outcomes (KDIGO) Diabetes Work Group, 2022), “National technical guidelines for the prevention and treatment of diabetic kidney disease in primary care (2023)” (Chinese Diabetes Society, 2023), “Guideline for the prevention and treatment of type 2 diabetes mellitus in China (2020 edition)” (Chinese Diabetes Society, 2021), “Guideline for the Management of Diabetes Mellitus in the Elderly in China (2024 edition)” (National Center of Gerontology et al., 2024), “Chinese guidelines of medical nutrition therapy in diabetes (2022 edition)” (Nutrition and Metabolic Management Branch of China et al., 2022), “Guideline for exercise therapy of type 2 diabetes mellitus in China (2024 edition)” (Gerontology. N C o et al., 2024).

There is often a significant gap between knowledge and action, and merely providing individuals with health knowledge is insufficient to promote behavior change. While many individuals may understand the correct practices, they often struggle to implement them in their daily lives. The theory addresses this gap by identifying the key factors that influence behavior. It offers a scientific framework and systematic approach to designing more effective interventions. These five topics are structured based on the theoretical framework of the HBM, which aims to enhance individuals' awareness of the severity of DKD, their susceptibility to it, and the benefits of making behavioral changes. By doing so, the program can stimulate patients' self-efficacy, encourage stronger self-management, and enhance the sustainability of behavior change. This theory-based intervention provides strong support for achieving individual health goals. The detailed Self-Management Education Program in Table 1.

**Table 1** **Self-Management** **Education Program**

| **Topic** | **Content** | **HBM based construct** |
| --- | --- | --- |
| DKD knowledge | Explanation of DKD, its development, influencing factors, health impacts, and psychological & Economic burdens. Assessment of patient's DKD modifiable factors and set a personalized control goals with feedback table. | Let patients perceive the seriousness of DKD and their own susceptibility to DKD, trigger patients' motivation to focus on self-management, provide support, and give patients confidence. |
| Diet Management | The first session includes assessing nutritional status, determining daily total calorie requirements, food categorization, planning diet structures, and ensuring a balanced diet. The second session of diet management includes the following contents: cooking methods, the sequence of food during meals, selection and conditions of consuming fruits, and individual dietary preferences. | Helping patients perceive the benefits of a balanced diet, assessing barriers to maintaining a balanced diet, and providing strategies to overcome them, instilling confidence in patients that they have the ability and resources for diet management. |
| Physical Activity | Includes self-preparation before exercise, types of exercise, duration and frequency of exercise, and prevention of hypoglycemia after exercise. | Enabling patients to perceive the benefits of regular exercise, assessing barriers and difficulties in their daily activity, providing alternative methods, and fostering the belief in patients that they can maintain a regular exercise routine. |
| Medication Management | Medication Management involves instructing patients on the types of antidiabetic medications, methods of administration, and precautions. For patients using insulin, providing a standardized injection procedure, and distributing a rotation card for injection sites is part of the intervention. The goal is to facilitate patients in following medical advice and using medications appropriately. | Enabling patients to perceive the benefits of following prescribed medications and understanding the severity of non-adherence. Addressing barriers to proper medication adherence, such as unfamiliarity with types of medications, dosing methods, and improper insulin injections. |
| Self-Monitoring | Includes  standardized procedures for blood glucose measurement and blood pressure measurement, to assist patients in effectively monitoring and control these well. | Helping patients recognize the benefits of self-monitoring, overcoming obstacles associated with self-monitoring, encouraging patients to strive towards established goals, and fostering patients' self-efficacy. |

Compared to standard care, the SMEP is more structured and comprehensive, differences between standard care and SMEP are detailed in Table 2.

**Table 2 Differences between standard care and intervention**

| **Items** | **Standard Care** | **Intervention** |
| --- | --- | --- |
| Emphasis | Emphasis on treatment and health. | Focus on education and empowerment, enabling individuals to understand their conditions and actively engage in self-management. |
| Framework | Encourage patients to change their current lifestyle to promote health. | Based on the framework of behavior change theory, scientific methods are used to more effectively facilitate patients in making actual changes. |
| DKD knowledge | Patients with T2DM who do not have DKD will not receive education about DKD knowledge. They are only be informed that there is a risk of developing kidney complications if blood glucose is not well controlled. | Patients will receive DKD knowledge. And assess the current risk factors for DKD and set a personalized goals for controlling these risk factors. |
| Diet management | The primary nurse educates patients about the glycemic index of common foods and advises on proportions of protein, carbohydrates, and vegetables. | 1. Helping patients perceive the benefits of a balanced diet.  2. Diet education encompasses a comprehensive approach, including the assessment of current nutritional status, calculation of total energy requirements, guidance on food selection and pairing, managing fruit intake.  3.Emphasizes the combined technique of plate apportion and eating sequence, while also considering the patient's preferences and dynamically adjusting total energy intake. |
| Physical Activity | The responsible nurse advises patients to take a leisurely walk after meals and avoid exercising on an empty stomach. | 1. Helping patients perceives the benefits of exercise.  2. Considering the patient's work and preferences, a suitable exercise method, frequency, and timing are tailored.  3. Educate patients on strategies to prevent hypoglycemia during exercise. |
| Medication Management | The responsible nurse informs patients about taking medication as prescribed by the doctor and the precautions for taking medication. | 1. Helping patients perceives the benefits of adhering to prescribed medication and the severity of non-adherence.  2. A specialized seven-compartment diabetes medication box is provided to prevent missed or incorrect doses. |
| Self-Monitoring | The responsible nurse informs the patient about the regular monitoring of blood glucose, blood pressure, and body weight, and teaches how to perform the measurements. | 1. The patient is informed about the benefits of self-monitoring and the potential risks associated with not understanding fluctuations in blood glucose levels.  2. Providing standardized blood glucose monitoring, documenting numerical changes, and encouraging patients to strive towards established goals. |
| Follow-up | The doctor advised a follow-up visit in six months for patients. | Monthly calls to monitor patients' proactive involvement in self-management and at the six-month mark, a phone notification is provided for a follow-up visit. |

**6.3 Intervention procedures**

The recruitment poster is placed at the nurses' station for patient enrollment. Potential participants who are interested in the study will be evaluated by a researcher to determine whether they meet the inclusion criteria. Participants who meet the criteria and sign the informed consent form will receive an envelope. The number on the envelope indicates the patient’s identification numbers refers to the enrollment sequence number, while the number inside the envelope determines their group allocation. After enrollment, the intervention will be carried out by a different researcher.

The intervention comprises two periods: a six-week initial learning phase, followed by the strengthening phase, which repeats the content from the first phase. The initial learning phase consists of six sessions, held once a week. The first session is scheduled during hospitalization, where on-site lectures are conducted using slides. As the hospitalization duration for diabetes patients is typically around one week, subsequent sessions are arranged after discharge. To improve convenience and participation rates, videos on the designated topics will be provided for participants to view on their own. Additionally, participants can pose questions after watching the video, and the researcher will provide answers.

The strengthening phase focuses on repeating and consolidating the knowledge and skills participants acquired during the initial phase. It aims to further promote the sustainability of behavior change, ensuring the internalization and practical application of knowledge and behaviors, ultimately supporting participants in achieving long-term health behavior changes.

Following the completion of the intervention, participants in the intervention group undergo a 12 weeks follow-up. Researchers conduct monthly telephone (or WeChat) calls to supervise patients, ensuring the continuation and integrity of self-management practices and promoting the formation of healthy habits. At the end of the follow-up period, patients are notified via telephone to return for a follow-up visit (As shown in Table 3).

**Table 3 Intervention implementation schedule**

| **Week** | **Time** | **Topic** | **Method** |
| --- | --- | --- | --- |
| 1 | During the hospitalization | DKD knowledge | Present a lecture using slides  30min |
| 2 | 2nd week after enrollment | Diet Management-Part 1 | Video and discussion online  20-30min |
| 3 | 3rd week after enrollment | Diet Management- Part 2 | Video and discussion online  20-30min |
| 4 | 4th week after enrollment | Physical Activity | Video and discussion online  20-30min |
| 5 | 5th week after enrollment | Medication Management | Video and discussion online  20-30min |
| 6 | 6th week after enrollment | Self-Monitoring | Video and discussion online  20-30min |
| 7~12 | Once a week | Strengthening Phase | Video and discussion online |
| Intervention ends | | | |
| 13~24 | once a month | Follow-up | Telephone (or WeChat) calls |

6.4 Control group

The control group received routine care provided by the responsible nurse. Based on the physician’s medical orders, standard diabetes health education was delivered, including basic knowledge of diabetes and its complications, normal glucose ranges, recognition and management of hyperglycemia and hypoglycemia, general dietary advice, and recommendations for moderate physical activity. Medication instructions, including dosage, timing, precautions, and insulin injection guidance (if applicable).

7 Outcomes and outcome measures

7.1 Primary outcomes

The primary outcomes are clinical indicators, that covers Hemoglobin A1c (HbA1c), urine albumin-to-creatinine ratio (UACR), estimated glomerular filtration rate (eGFR), systolic blood pressure (SBP), low-density lipoprotein cholesterol (LDL-C), body mass index (BMI), waist circumference (WC).

7.2 Secondary outcomes

7.2.1 Self-Management behavior

Diabetes Self-Management Questionnaire (DSMQ) was developed by German scholars (Schmitt A et al., 2013), comprises 16 items and organized into four dimensions: glucose management, dietary control, physical activity, and health-care use, the Cronbach's α coefficient of the Sum Scale is 0.84. After being translated and culturally adapted by Chinese scholars (LI Chao-qun et al., 2018), the scale retained its 16 items but was restructured into five dimensions: blood glucose monitoring, adherence to medication, dietary control, physical activity, and follow-up. The scale uses a 4-point Likert scoring method. For items 1, 2, 3, 4, 6, 8, 9, which represent positive self-management behaviors, scores are assigned as follows: 3 for "Strongly Agree," 2 for "Agree," 1 for "Disagree," and 0 for "Strongly Disagree." For items 5, 7, 10, 11, 12, 13, 14, 15, and 16, which represent negative self-management behaviors, scores are assigned in reverse: 0 for "Strongly Agree," 1 for "Agree," 2 for "Disagree," and 3 for "Strongly Disagree." The score is normalized using the formula: actual score = raw score / theoretical maximum score * 10. The total score ranges from 0 to 10, with a higher score indicating stronger self-management abilities. The Cronbach's α coefficient of the total scale is 0.84, with the Cronbach's α coefficients for the subscales ranging from 0.60 to 0.77.

7.2.2 Diabetes-related Knowledge level

The Diabetes Knowledge Test (DKT) was originally developed by the Michigan Diabetes Research and Training Centre (Fitzgerald J T et al., 1998). The scale comprises two components: a 14-item general knowledge test and a 9-item insulin-use subscale. Subsequently, the original authors refined the wording and grammar of the scale without adding or removing any items. The revised version underwent validation, demonstrating reliability with Cronbach's α of 0.77 for the general test and 0.84 for the insulin-use subscale (Fitzgerald J T et al., 2016). The scale was culturally adapted by Chinese researchers to better align with local dietary habits (Wang Rui et al., 2023). DKT is also a short test. Each correct answer is awarded one point, with a maximum score of 14 for the general test, and 23 for all items tests. Standardized scoring is applied to ensure comparability of knowledge scores between patients who use insulin and those who do not. The standardized score is calculated using the formula: standardized score =(raw score/ maximum possible score)×10, higher scores indicate a better understanding of diabetes-related knowledge. DKT demonstrates strong reliability, with a Cronbach’s α coefficient of 0.806, a split-half reliability of 0.739, and a test-retest reliability of 0.745. Specifically, the general test had a Cronbach’s α coefficient of 0.762, while the insulin-related knowledge test had a Cronbach’s α coefficient of 0.812.

7.2.3 Self-efficacy level

Self-Efficacy for Diabetes (SED) scale originally developed by Lorig K and colleagues during their research on diabetes self-management, and later updated by the Stanford University Research Centers (Stanford University Research Centers., 2025). The original scale comprises eight items, each rated on a scale from 1 (not at all confident) to 10 (totally confident), with the total score being the mean of all items, the scale demonstrates good internal consistency reliability, with a Cronbach's α of 0.828. The scale was translated into Chinese and culturally adapted (Jie W, 2013). The revised Chinese version consists of 9 items that reflect patients' self-efficacy in five aspects: diet, exercise, glucose management, and disease control. It uses a 5-point Likert scale, with responses scored from 1 to 5. These scores correspond to levels of confidence ranging from "completely lacking confidence" to "lacking confidence", "somewhat confident", "very confident" and "completely confident. The total score is the mean of 9 items scores, ranges from 1 to 5, higher scores indicate better self-efficacy in patients. The Cronbach's α for the scale is 0.890.

7.2.4 Perception of Risk of Chronic Kidney Disease

Perception of Risk of Chronic Kidney Disease Scale (PRCKDS) was developed by Chinese scholars (Xia C et al., 2022). This questionnaire aims to evaluate the risk perception of Chronic Kidney Disease in patients with type 2 diabetes, encompassing five dimensions: susceptibility, severity, benefits, barriers, and triggering factors. It comprises a total of 36 items rated on a Likert scale of 4 levels. Response options include " completely disagree," "disagree," "agree," and "strongly agree," corresponding to scores of 1 to 4 respectively. The items in the barriers dimension are reverse scored, with the scoring rule being 5 minus the original score. Scores for each dimension are converted to a 10-point scale to facilitate interdimensional comparisons, using the formula: dimension score / maximum possible score for the dimension × 10, the minimum score for each dimension is 2.5 points, and the maximum score is 10 points. After rescaling, the total score for the scale is computed as the sum of scores across all dimensions, the score of this scale ranges from 12.5 to 50. A higher score indicates a higher level of perceived risk of Chronic Kidney Disease. The Cronbach's α coefficient for the scale is 0.875, and the split-half reliability is 0.865.

8 Data collection

Two researchers, who are not involved in group assignment, collect and record participants’ data based on identification numbers refers to the enrollment sequence number. The researcher initially collect data after the participants are enrolled in the group (baseline, T0), the second time at the end of intervention (12 weeks after enrollment, T1), and the third data collection conducted at the end of follow-up (24 weeks after enrollment, T2). Participants independently completed questionnaires, while laboratory indicators were recorded by researchers based on medical reports. For individuals who were unable to independently complete the questionnaire, researchers conducted one-on-one interviews.

9 Statistical analysis

Using SPSS 29.0 statistical software for data analysis. For continuous data, normality testing is conducted first. Descriptive statistics are presented as mean ± standard deviation for data following a normal distribution and as median (interquartile range) for non-normally distributed data. Categorical data are described using frequencies and percentages. Comparison between two groups: For normally distributed continuous data, a t-test is employed; for non-normally distributed data, non-parametric tests are used. Categorical data are analyzed using the chi-square test or Fisher's exact test. The analysis of this study will follow the intention-to-treat (ITT) principle. Generalized Estimating Equations (GEE) model were applied to assess the effectiveness of the intervention over time, accounting for within-subject correlations among repeated measurements. A significance level of P < 0.05 is considered statistically significant.
