# Supplementary material for: Effectiveness of a self-management intervention on clinical outcomes among patients with type 2 diabetes at high risk for Diabetic Kidney Disease: A Randomised Controlled Trial
Source: PLoS One. 2026 Oct 12;21(10):e0360475. doi: 10.1371/journal.pone.0360475 (PMC13657325; doi:10.1371/journal.pone.0360475)
Supplement: Supplementary file 4 [file pone.0360475.s002.docx]

**Technical Review Application**

## 1. Study Title

Effectiveness of Self-Management Education in Patients with Type 2 Diabetes at High Risk of Diabetic Kidney Disease

## 2. Study Background and Objectives

With the continuous increase in the prevalence of type 2 diabetes mellitus (T2DM), the number of patients with diabetic kidney disease (DKD) is also rising rapidly. DKD is one of the most common microvascular complications of T2DM and has a significant negative impact on patients. The early stages of DKD are often asymptomatic, and clinical diagnosis is typically made at stage 3. At this point, slowing disease progression largely depends on pharmacological treatment and renal replacement therapy. Therefore, early prevention of DKD is of greater importance than treatment.

However, patients with T2DM often lack awareness of DKD risk and a proper understanding of the disease. Identifying individuals at high risk of DKD among patients with T2DM and implementing targeted interventions at this stage can prevent or delay the onset of DKD, reduce related complications and healthcare costs, and ultimately alleviate the long-term burden on patients.

A retrospective cohort study reported that, compared with participants adhering to 0–1 low-risk lifestyle behaviors, patients with diabetes who adhered to 4–5 low-risk lifestyle behaviors had a 57% lower risk of developing chronic kidney disease.

Through self-management interventions, this study aims to improve risk awareness and understanding of DKD among high-risk individuals, encourage active participation in health management, promote healthy lifestyles, improve control of risk factors, and ultimately reduce the incidence of DKD. The findings may provide a reference for the future management of complications in patients with T2DM.

## 3. Study Design and Methods

### 3.1 Participant Selection Criteria

#### 3.1.1 Inclusion Criteria

1. Patients meeting the World Health Organization diagnostic criteria for T2DM;
2. Patients at high risk for DKD;
3. Aged 39–75 years;
4. Able to use WeChat;
5. Willing to participate and provide written informed consent.

#### 3.1.2 Exclusion Criteria

1. Patients with a confirmed diagnosis of DKD;
2. Patients with mental disorders or cognitive impairment who are unable to cooperate with the study;
3. Secondary glomerular diseases caused by primary renal disease, hypertension, or renal arteriosclerosis;
4. Recent conditions affecting urinary protein levels, such as diabetic ketoacidosis, acute nephritis, or urinary tract infection.

## 3.2 Intervention

### 3.2.1 Control Group

Participants in the control group will receive routine care provided by primary nurses, including admission education, routine nursing care, and health education. The content covers medication management, hypoglycemia management, dietary guidance, exercise guidance, and discharge instructions. Patients are generally advised to attend follow-up visits every six months.

### 3.2.2 Intervention Group

In addition to routine care, participants in the intervention group will receive a structured self-management education program delivered by the research team. The program includes five core components:1.DKD knowledge；2.Dietary management；3.Physical activity；4.Medication management；5.Self-monitoring.

These components are structured based on the Health Belief Model (HBM), including perceived susceptibility, perceived benefits, perceived barriers, perceived severity, cues to action, and self-efficacy.

In this program, patients are encouraged to recognize the severity of DKD and their susceptibility to it. To reduce the risk of disease progression, patients are guided to understand the benefits of self-management. By enhancing self-efficacy, patients are supported in overcoming barriers and maintaining effective self-management behaviors. This approach aims to motivate individuals and strengthen their commitment to adopting positive health behaviors.

According to the American Diabetes Association Professional Practice Committee, self-management interventions lasting more than 6 months and totaling more than 10 hours are associated with better outcomes. In this study, participants in the intervention group will receive a 12-week self-management education program followed by a 12-week follow-up.

The intervention includes 12 sessions covering the five topics (with dietary management divided into two sessions), delivered by the research team, along with 6 additional sessions focusing on patient interaction and experience sharing.

Participants will be invited to join a WeChat group for communication and management. The first session will be conducted face-to-face during hospitalization using presentation slides. Subsequent sessions will be conducted after discharge. To improve accessibility and participation, video materials will be provided for self-paced learning. After watching the videos, participants will engage in online group discussions via WeChat, where they can ask questions and receive guidance from researchers.

Sessions 7–12 will revisit the initial topics. In addition, experience-sharing meetings will be organized to provide a supportive environment for participants to share daily experiences, challenges, and successes, promoting mutual learning and engagement.

After the intervention, participants will undergo a 12-week follow-up period. Researchers will conduct monthly follow-ups via telephone or WeChat to monitor adherence, reinforce self-management behaviors, and support the maintenance of healthy habits. At the end of follow-up, participants will be contacted to schedule return visits.

## 3.3 Outcome Measures

### Primary Outcomes

Clinical indicators, including:

- Hemoglobin A1c (HbA1c)
- Urinary albumin excretion rate (UAER)
- Estimated glomerular filtration rate (eGFR)
- Systolic blood pressure (SBP)
- Low-density lipoprotein cholesterol (LDL-C)
- Body mass index (BMI)
- Waist circumference (WC)

### Secondary Outcomes

Psychosocial measures, including:

- Self-management behavior
- Diabetes-related knowledge
- Self-efficacy
- Risk perception of chronic kidney disease

## 4. Data Management and Statistical Analysis

All data will be recorded in accordance with study requirements, and missing data will be documented appropriately to ensure data accuracy and integrity.

Statistical analyses will be performed using SPSS version 26.0. Continuous variables will be expressed as mean ± standard deviation. For normally distributed data, t-tests will be used (paired t-tests for within-group comparisons and independent t-tests for between-group comparisons). For non-normally distributed data, non-parametric tests will be applied. Categorical variables will be analyzed using the chi-square test, and ordinal data will be analyzed using rank-sum tests. Generalized estimating equations (GEE) will be used to evaluate intervention effects. A p-value < 0.05 will be considered statistically significant.

## 5. Ethical Considerations

### 5.1 Ethical Approval

Before the initiation of the study, the study protocol, jointly reviewed and signed by the investigators and sponsor, must be submitted to the Institutional Ethics Committee for approval. If modifications are required during the study, they must be reviewed and approved by the coordinating committee and resubmitted to the Ethics Committee before implementation.

### 5.2 Informed Consent

Before enrollment, participants will be fully informed about the study, including its purpose, procedures, potential benefits and risks, and their rights and obligations. Written informed consent will be obtained from all participants. Participants’ contact information will be recorded, and researchers will provide contact details to facilitate communication and follow-up.

### 5.3 Risk–Benefit Assessment

Potential benefits include improved self-management ability and possible improvements in clinical outcomes. Potential risks include inconvenience associated with follow-up. Researchers may terminate participation if necessary to protect participants’ health and safety.

### 5.4 Privacy Protection

Only authorized research personnel will have access to participants’ medical records. Confidentiality will be strictly maintained. Data will be anonymized during analysis to remove identifiable information. All records will be securely stored in designated clinical research facilities.
