# Supplementary material for: Effectiveness of a self-management intervention on clinical outcomes among patients with type 2 diabetes at high risk for Diabetic Kidney Disease: A Randomised Controlled Trial
Source: PLoS One. 2026 Oct 12;21(10):e0360475. doi: 10.1371/journal.pone.0360475 (PMC13657325; doi:10.1371/journal.pone.0360475)
Supplement: Supplementary file 5 [file pone.0360475.s003.docx]

**English Translation of Ethics Approval**

Zaozhuang Central Hospital of Shandong Guoxin Yiyang Group

Medical Ethics Committee

**Ethics Review Approval**

Ethics Approval No.: 2024-Ethics Review-015

| Date of Ethical Approval | October 18, 2024 |
| --- | --- |
| **Title of the Study** | Effectiveness of a Self-Management Education Program among Individuals at High Risk of Diabetic Kidney Disease |
| **Sponsor** | Zaozhuang Central Hospital of Shandong Guoxin Healthcare Group |
| Department / Principal Investigator | Department of Endocrinology /Han Xinjian, Chu Dongmei |
| Type of Review | Initial review |
| Review Method | Meeting review |
| Documents Reviewed | 1.Submission letter  2.Clinical research protocol  3.Informed consent form |
| Meeting Attendance: Members required: 15, Members present: 13, Members recused: 1. | |
| **Voting Results:** Approved: 12, Approved with modification: 0, Not approved: 0. | |
| Ethics Committee Opinion:  The Medical Ethics Committee reviewed the research protocol and informed consent documents on October 18, 2024. The study protocol (Version No.: 2023-16BDY-12-V02, Version Date: 20240419) was reviewed and approved as it complies with ethical requirements. The study is approved to be conducted. Researchers are required to strictly comply with Good Clinical Practice (GCP) guidelines and the Declaration of Helsinki.  Continuing Review Interval: 12 months | |
| Precautions:  1. Any modification to the study materials during the trial must be submitted to the Ethics Committee for review and approval.  2. Any Suspected Unexpected Serious Adverse Reaction (SUSAR) that is fatal or life-threatening must be reported within 7 days of awareness, followed by a complete follow-up report within the subsequent 8 days. Non-fatal or non–life-threatening SUSARs should be reported within 15 days of initial awareness.  3. Any protocol deviation or violation occurring during the study must be promptly reported by submitting a Protocol Deviation/Violation Report.  4. According to the required periodic continuing review schedule, a continuing review application and report must be submitted to the Ethics Committee one month prior to the review date.  5. From the date of approval, an annual progress report must be submitted to the Ethics Committee every year. The application and report for annual review should be submitted one month prior to the review date.  6. In the event of study suspension or premature termination, an application for suspension or termination review must be submitted in a timely manner.  7. Upon completion of the study, a final study report must be submitted to the Ethics Committee. | |
| Validity Period | October 18, 2024 – October 17, 2025 |
| Contact Person/Telephone | Zong Zhaoyan/0632-4060134 |
| Chairperson Signature **Date:** October 18, 2024 | |
| Zaozhuang Central Hospital of Shandong Guoxin Yiyang Group Medical Ethics Committee (Official Seal) | |

Translation statement

This document is an English translation of the original ethics approval issued in Chinese. The translation was prepared by the authors, and the content is consistent with the original document.
