# Supplementary material for: Effectiveness of a self-management intervention on clinical outcomes among patients with type 2 diabetes at high risk for Diabetic Kidney Disease: A Randomised Controlled Trial
Source: PLoS One. 2026 Oct 12;21(10):e0360475. doi: 10.1371/journal.pone.0360475 (PMC13657325; doi:10.1371/journal.pone.0360475)
Supplement: Supplementary file 7 [file pone.0360475.s005.docx]

**S5 Table. Consistency of results between generalized estimating equations analyses based on original (non-imputed) data and multiple imputation analyses, including group-by-time interactions and between-group differences at each time point**

| **Outcome** | **Effect** | **Original data** | | **Imputed data** | | **Consistency** |
| --- | --- | --- | --- | --- | --- | --- |
|  |  | **Wald χ²** | **P** | **Wald χ²** | **P** |  |
| HbA1c | Group × time | 9.693 | 0.008 | 8.701 | 0.013 | Consistent |
|  | Between-group difference (T0) | 1.056 | 0.304 | 0.996 | 0.318 | Consistent |
|  | Between-group difference (T1) | 8.508 | 0.004 | 7.866 | 0.005 | Consistent |
|  | Between-group difference (T2) | 13.813 | <0.001 | 12.767 | <0.001 | Consistent |
| UACR | Group × time | 151.087 | <0.001 | 97.071 | <0.001 | Consistent |
|  | Between-group difference (T0) | 0.503 | 0.478 | 0.491 | 0.483 | Consistent |
|  | Between-group difference (T1) | 2.179 | 0.140 | 2.039 | 0.153 | Consistent |
|  | Between-group difference (T2) | 2.931 | 0.087 | 2.972 | 0.085 | Consistent |
| eGFR | Group × time | 29.047 | <0.001 | 11.603 | 0.003 | Consistent |
|  | Between-group difference (T0) | 1.392 | 0.238 | 1.408 | 0.235 | Consistent |
|  | Between-group difference (T1) | 1.428 | 0.232 | 1.456 | 0.228 | Consistent |
|  | Between-group difference (T2) | 4.479 | 0.034 | 4.117 | 0.042 | Consistent |
| LDL-C | Group × time | 12.140 | 0.002 | 12.627 | 0.002 | Consistent |
|  | Between-group difference (T0) | 3.412 | 0.065 | 3.370 | 0.066 | Consistent |
|  | Between-group difference (T1) | 9.879 | 0.002 | 9.960 | 0.002 | Consistent |
|  | Between-group difference (T2) | 15.935 | <0.001 | 16.608 | <0.001 | Consistent |
| SBP | Group × time | 351.386 | <0.001 | 280.744 | <0.001 | Consistent |
|  | Between-group difference (T0) | 1.232 | 0.267 | 1.225 | 0.268 | Consistent |
|  | Between-group difference (T1) | 2.983 | 0.084 | 2.765 | 0.096 | Consistent |
|  | Between-group difference (T2) | 4.847 | 0.028 | 4.975 | 0.026 | Consistent |
| BMI | Group × time | 25.029 | <0.001 | 16.448 | <0.001 | Consistent |
|  | Between-group difference (T0) | 0.892 | 0.345 | 0.877 | 0.349 | Consistent |
|  | Between-group difference (T1) | 0.038 | 0.845 | 0.035 | 0.851 | Consistent |
|  | Between-group difference (T2) | 0.072 | 0.789 | 0.086 | 0.769 | Consistent |
| WC | Group × time | 0.702 | 0.704 | 0.395 | 0.821 | Consistent |
|  | Between-group difference (T0) | 1.449 | 0.229 | 1.417 | 0.234 | Consistent |
|  | Between-group difference (T1) | 0.804 | 0.370 | 0.776 | 0.378 | Consistent |
|  | Between-group difference (T2) | 0.844 | 0.358 | 0.956 | 0.328 | Consistent |
