# Supplementary material for: Effectiveness of a self-management intervention on clinical outcomes among patients with type 2 diabetes at high risk for Diabetic Kidney Disease: A Randomised Controlled Trial
Source: PLoS One. 2026 Oct 12;21(10):e0360475. doi: 10.1371/journal.pone.0360475 (PMC13657325; doi:10.1371/journal.pone.0360475)
Supplement: Supplementary file 8 [file pone.0360475.s006.docx]

**Figure 1. Changes in HbA1c over time in the intervention and control groups (mean ± 95% CI) based on GEE models**

**Figure 2. Changes in UACR over time in the intervention and control groups (mean ± 95% CI) based on GEE models**

 **Figure 3. Changes in eGFR over time in the intervention and control groups (mean ± SE) based on GEE models**

 **Figure 4. Changes in SBP over time in the intervention and control groups (mean ± SE) based on GEE models**

 **Figure 5. Changes in LDL-C over time in the intervention and control groups (mean ± SE) based on GEE models**

 **Figure 6. Changes in BMI over time in the intervention and control groups (mean ± SE) based on GEE models**

 **Figure 7. Changes in WC over time in the intervention and control groups (mean ± SE) based on GEE models**
