# Supplementary material for: Effectiveness of a self-management intervention on clinical outcomes among patients with type 2 diabetes at high risk for Diabetic Kidney Disease: A Randomised Controlled Trial
Source: PLoS One. 2026 Oct 12;21(10):e0360475. doi: 10.1371/journal.pone.0360475 (PMC13657325; doi:10.1371/journal.pone.0360475)
Supplement: Supplementary file 11 [file pone.0360475.s012.docx]

**Response to Reviewers**

Dear Editor and Reviewers,

We sincerely thank you for your valuable comments. We carefully reviewed all the comments and revised the manuscript where appropriate. Detailed responses are provided below.

**Abstract：** Thank you for your valuable comments. We revised the Abstract to clarify the definition of high-risk DKD, improve the description of the intervention, correct the participant attrition information, and strengthen the practical implications in the Conclusion. Considering the Abstract word limit, detailed statistical results remain presented in the Results section.

**Introduction：**Thank you for your valuable comments. We carefully reviewed the Introduction and confirmed that the current version already addresses the disease burden, rationale for early intervention, existing evidence, research gap, and study objectives. Therefore, the current text has been retained.

**Methods：**Thank you for your valuable comments. We carefully reviewed the Methods section and confirmed that the current manuscript already provides sufficient information regarding the study design, randomization, blinding, sample size calculation, participant eligibility, intervention, missing data handling, ethical approval, and trial registration. Therefore, the current text has been retained.

**Results and Tables**：Thank you for your valuable comments. We carefully reviewed the Results and tables. The participant flow numbers were verified for consistency, and the CONSORT flow diagram is provided as a separate uploaded file. Baseline characteristics are presented in **Table 3**, and adjusted estimates, 95% confidence intervals, and within-group and between-group comparisons are presented in **Table 4**. Statistical values and p values are consistently reported to three decimal places, and detailed sensitivity analysis results are provided in **Supplementary File S5**.

**Discussion：** We carefully reviewed the Discussion and confirmed that the current manuscript already addresses the main findings, their interpretation, comparisons with previous studies, and the implications for DKD prevention. We revised the **Implications for Clinical Practice** section to further emphasize the feasibility and scalability of the video-based intervention and revised the **Conclusion** to provide a more balanced summary of the study findings.
