# Supplementary material for: Effectiveness of a self-management intervention on clinical outcomes among patients with type 2 diabetes at high risk for Diabetic Kidney Disease: A Randomised Controlled Trial
Source: PLoS One. 2026 Oct 12;21(10):e0360475. doi: 10.1371/journal.pone.0360475 (PMC13657325; doi:10.1371/journal.pone.0360475)
Supplement: Supplementary file 12 [file pone.0360475.s010.docx]

**Response to Reviewers**

Dear Editor,

Thank you for your comments and the detailed requirements. We have carefully addressed all the points as requested.

Specifically, the manuscript has been revised to meet PLOS ONE formatting and style requirements. The completed “Inclusivity in Global Research” questionnaire has been uploaded as Supporting Information. The minimal dataset required to replicate the study findings has been provided in the Supporting Information files. The ORCID iD for the corresponding author has been updated and validated in Editorial Manager. All Supporting Information files have been properly organized, and their captions have been added to the end of the manuscript with corresponding in-text citations updated accordingly. In addition, the text has been translated into English in accordance with requirements.

We hope that the revised manuscript meets the journal’s standards and look forward to your feedback.

**Response to reviewer 1**

Dear Reviewer,

Thank you for these valuable comments. We have carefully revised the manuscript accordingly, and the main revisions are summarized below.

1. We have now explicitly specified HbA1c as the primary endpoint in the revised manuscript. HbA1c was selected because it is the most widely accepted and clinically relevant indicator of long-term glycemic control in patients with diabetes and serves as a key target in diabetes management guidelines. In addition, HbA1c is closely associated with the risk of developing and progressing diabetic kidney disease. While renal outcomes such as UACR and eGFR are also clinically important, they were considered secondary endpoints to provide complementary information on renal function and disease progression. Given that HbA1c was pre-specified as the primary endpoint and the study was powered based on this outcome, analyses of secondary outcomes were considered exploratory, and the results were interpreted with caution due to multiple comparisons. These clarifications have been incorporated into the revised manuscript.

2. The GEE model specification has been further clarified by explicitly stating the use of a normal distribution with an identity link function, and robust standard errors were applied in all analyses. In addition, skewed variables (HbA1c and UACR) were log-transformed prior to analysis, and the results were back-transformed for ease of interpretation. These revisions have been incorporated into the Methods (Section 2.8) and reflected in the corresponding Results section.

3. We have conducted sensitivity analyses using multiple imputation with 10 imputed datasets to assess the robustness of our findings to missing data. The imputed datasets were analyzed using the same GEE models, and the results were pooled using Rubin’s rules implemented in R. The findings from the multiple imputation analyses were consistent with those from the primary analyses based on the original data, indicating that the conclusions are robust to the handling of missing data under the assumption of missing at random. These details have been added to the Methods and Results sections, and the full results are presented in Supplementary Table S5.

4. The sample size was determined based on the primary outcome. Given the repeated-measures design and the use of GEE, the analysis benefits from increased statistical efficiency by accounting for within-subject correlation over time. In addition, we have clarified the analytic details, including the covariates (education level, family history of diabetes, diabetes duration, and DKD risk score) and the use of an exchangeable working correlation structure. Finally, graphical displays of estimated marginal means with 95% confidence intervals have been added in the Supplementary Materials.

5. We have revised the Results section following a stepwise, decision-guided process. For outcomes with a statistically significant group-by-time interaction, we removed the reporting and interpretation of the main effects (time and group), as these are not meaningful in the presence of a significant interaction. Instead, we focused on the interaction term and presented time-specific comparisons. For outcomes without a significant interaction, the main effects were retained and interpreted appropriately.

Thank you again for your helpful suggestions.

Kind regards,

**Response to reviewer 2**

Dear Reviewer,

Thank you for your valuable comments. The manuscript has been revised accordingly. The Introduction has been updated to clarify the research gap and study objectives. The Methods section has been further detailed, including clarification of the sample size calculation and randomization procedures. The tables have been revised, and figures have been added to present the trends of each outcome. The Discussion has been improved to present the potential mechanisms of the self-management program. The intervention effectively improved glycemic regulation, emphasized the importance of glycemic control in preventing DKD progression, and highlighted participants’ susceptibility to DKD. By increasing awareness of personal risk, the intervention promoted behavioral change and strengthened self-management adherence, which may have contributed to the observed improvements in metabolic outcomes. In addition, study limitations and clinical implications have been described in Section 4.2. Finally, the manuscript has been revised according to the journal requirements, with language editing, standardized terminology, and improved consistency. In accordance with guideline usage, “kidney” is used for disease-related terms (e.g., diabetic kidney disease), while “renal” is used for functional descriptions (e.g., renal injury, renal inflammation).

Thank you again for your helpful suggestions.

Kind regards,
