# Supplementary material for: Effectiveness of a self-management intervention on clinical outcomes among patients with type 2 diabetes at high risk for Diabetic Kidney Disease: A Randomised Controlled Trial
Source: PLoS One. 2026 Oct 12;21(10):e0360475. doi: 10.1371/journal.pone.0360475 (PMC13657325; doi:10.1371/journal.pone.0360475)
Supplement: Peer review history

# Peer review: Decision Letter 0

**DOI:** 10.1371/journal.pone.0360475.r001

**Authors:** Diego Moriconi

10 Mar 2026

Dear Dr.  Japar,

Thank you for submitting your manuscript to PLOS ONE. After careful consideration, we feel that it has merit but does not fully meet PLOS ONE’s publication criteria as it currently stands. Therefore, we invite you to submit a revised version of the manuscript that addresses the points raised during the review process.

Please submit your revised manuscript by Apr 24 2026 11:59PM. If you will need more time than this to complete your revisions, please reply to this message or contact the journal office at plosone@plos.org. When you're ready to submit your revision, log on to https://www.editorialmanager.com/pone/ and select the 'Submissions Needing Revision' folder to locate your manuscript file.

- A letter that responds to each point raised by the academic editor and reviewer(s). You should upload this letter as a separate file labeled 'Response to Reviewers'.
- A marked-up copy of your manuscript that highlights changes made to the original version. You should upload this as a separate file labeled 'Revised Manuscript with Track Changes'.
- An unmarked version of your revised paper without tracked changes. You should upload this as a separate file labeled 'Manuscript'.

If you would like to make changes to your financial disclosure, please include your updated statement in your cover letter. Guidelines for resubmitting your figure files are available below the reviewer comments at the end of this letter.

If applicable, we recommend that you deposit your laboratory protocols in protocols.io to enhance the reproducibility of your results. Protocols.io assigns your protocol its own identifier (DOI) so that it can be cited independently in the future. For instructions see: https://journals.plos.org/plosone/s/submission-guidelines#loc-laboratory-protocols. Additionally, PLOS ONE offers an option for publishing peer-reviewed Lab Protocol articles, which describe protocols hosted on protocols.io. Read more information on sharing protocols at https://plos.org/protocols?utm_medium=editorial-email&utm_source=authorletters&utm_campaign=protocols.

We look forward to receiving your revised manuscript.

Kind regards,

Diego Moriconi

Academic Editor

PLOS One

Journal Requirements:

When submitting your revision, we need you to address these additional requirements.

1.Please ensure that your manuscript meets PLOS ONE's style requirements, including those for file naming. The PLOS ONE style templates can be found at

https://journals.plos.org/plosone/s/file?id=wjVg/PLOSOne_formatting_sample_main_body.pdf and

https://journals.plos.org/plosone/s/file?id=ba62/PLOSOne_formatting_sample_title_authors_affiliations.pdf

2. Please include a complete copy of PLOS’ questionnaire on inclusivity in global research in your revised manuscript. Our policy for research in this area aims to improve transparency in the reporting of research performed outside of researchers’ own country or community. The policy applies to researchers who have travelled to a different country to conduct research, research with Indigenous populations or their lands, and research on cultural artefacts. The questionnaire can also be requested at the journal’s discretion for any other submissions, even if these conditions are not met.  Please find more information on the policy and a link to download a blank copy of the questionnaire here: https://journals.plos.org/plosone/s/best-practices-in-research-reporting. Please upload a completed version of your questionnaire as Supporting Information when you resubmit your manuscript.

3. We note that your Data Availability Statement is currently as follows: All relevant data are within the manuscript and in Supporting Information files.

Please confirm at this time whether or not your submission contains all raw data required to replicate the results of your study. Authors must share the “minimal data set” for their submission. PLOS defines the minimal data set to consist of the data required to replicate all study findings reported in the article, as well as related metadata and methods (https://journals.plos.org/plosone/s/data-availability#loc-minimal-data-set-definition).

For example, authors should submit the following data:

- The values behind the means, standard deviations and other measures reported;

- The values used to build graphs;

- The points extracted from images for analysis.

Authors do not need to submit their entire data set if only a portion of the data was used in the reported study.

If your submission does not contain these data, please either upload them as Supporting Information files or deposit them to a stable, public repository and provide us with the relevant URLs, DOIs, or accession numbers. For a list of recommended repositories, please see https://journals.plos.org/plosone/s/recommended-repositories.

If there are ethical or legal restrictions on sharing a de-identified data set, please explain them in detail (e.g., data contain potentially sensitive information, data are owned by a third-party organization, etc.) and who has imposed them (e.g., an ethics committee). Please also provide contact information for a data access committee, ethics committee, or other institutional body to which data requests may be sent. If data are owned by a third party, please indicate how others may request data access.

4. PLOS requires an ORCID iD for the corresponding author in Editorial Manager on papers submitted after December 6th, 2016. Please ensure that you have an ORCID iD and that it is validated in Editorial Manager. To do this, go to ‘Update my Information’ (in the upper left-hand corner of the main menu), and click on the Fetch/Validate link next to the ORCID field. This will take you to the ORCID site and allow you to create a new iD or authenticate a pre-existing iD in Editorial Manager.

5. Please include captions for your Supporting Information files at the end of your manuscript, and update any in-text citations to match accordingly. Please see our Supporting Information guidelines for more information: http://journals.plos.org/plosone/s/supporting-information.

6. Thank you for providing your underlying data as Supporting Information.

We note that the data set contains text or data that is not in English. Please note that PLOS is an English-language publisher, so we require data sets to be provided in English as well. Please upload an English-language version of your data set.

This will also allow us to determine if your data follows PLOS standards per our Data Availability policy here: https://journals.plos.org/plosone/s/data-availability.

7. If the reviewer comments include a recommendation to cite specific previously published works, please review and evaluate these publications to determine whether they are relevant and should be cited. There is no requirement to cite these works unless the editor has indicated otherwise.

Additional Editor Comments:

Dear Authors,

Thank you for submitting your manuscript to our journal. After careful evaluation and consideration of the reviewers’ reports, we have reached a decision regarding your submission.

The reviewers recognize the potential interest and relevance of your work. However, they have also identified several issues that need to be addressed before the manuscript can be considered for publication

Reviewers' comments:

Reviewer's Responses to Questions

**Comments to the Author**

1. Is the manuscript technically sound, and do the data support the conclusions?

Reviewer #1: Partly

Reviewer #2: Yes

**********

2. Has the statistical analysis been performed appropriately and rigorously?

Reviewer #1: Yes

Reviewer #2: Yes

**********

3. Have the authors made all data underlying the findings in their manuscript fully available?

The PLOS Data policy

Reviewer #1: Yes

Reviewer #2: Yes

**********

4. Is the manuscript presented in an intelligible fashion and written in standard English?

Reviewer #1: Yes

Reviewer #2: No

**********

Reviewer #1: This randomized controlled trial evaluated a structured, theory‑based self‑management program for adults with type 2 diabetes at high risk of developing diabetic kidney disease. The 24‑week intervention significantly improved HbA1c, albuminuria (UACR), blood pressure, LDL‑cholesterol, and slowed the decline in kidney function compared with usual care. No meaningful differences were found for BMI or waist circumference. Overall, the program effectively modified key risk factors that contribute to diabetic kidney disease progression.

Minor revisions:

1. Declare a primary endpoint (e.g., UACR or HbA1c) and apply an appropriate multiplicity‑control procedure for all secondary outcomes.

2. Specify the GEE model details, including the distribution family and link function; use robust standard errors, and appropriately transform skewed variables (such as applying a log‑transformation to UACR) while reporting back‑transformed estimates for interpretability.

3. Perform missing‑data sensitivity analyses—such as multiple imputation or pattern‑mixture models—to evaluate whether conclusions remain robust beyond the assumption of missing at random (MAR).

4. Provide a concise power justification tailored to the repeated‑measures design, including assumptions about within‑subject correlation and the expected group‑by‑time interaction, and specifically discuss whether the study has adequate power to detect changes in renal outcomes. Also include pre‑registered analytic details (covariates, correlation structure) and add graphical displays of marginal means with confidence intervals.

5. When evaluating interactions, interpret results only if the interaction term is significant, and avoid testing main effects in that case because they are not meaningful. If the interaction is not significant, remove it from the model and then test the main effects. Recognize that determining which results to present in the presence of interactions is a stepwise, decision‑guided process.

Reviewer #2: The manuscript addresses an important clinical issue by evaluating a structured self-management intervention for type 2 diabetes patients at high risk of diabetic kidney disease. The study design, a randomized controlled trial—is appropriate, and the results provide valuable insights into improvements in key clinical outcomes such as HbA1c, UACR, eGFR, SBP, and LDL-C. However, several areas require revision to enhance clarity, rigor, and reproducibility. The introduction would benefit from a clearer statement of the research gap and explicit study objectives, supported by updated literature on DKD and early prevention strategies. The methodology should provide more detail on participant recruitment, inclusion and exclusion criteria, ethical approvals, randomization procedures, intervention components, and statistical analysis assumptions. Results presentation could be improved with clearer tables and figures, effect sizes, confidence intervals, and a focus on clinically meaningful changes. The discussion should better contextualise findings within existing literature, explore potential mechanisms, acknowledge study limitations, and outline practical implications for patient care and clinical practice. Finally, attention to formatting, consistency, and language editing will enhance readability. Addressing these points will strengthen the manuscript and improve its contribution to the field of diabetes care and early DKD prevention.

**********

what does this mean?). If published, this will include your full peer review and any attached files.

If you choose “no”, your identity will remain anonymous but your review may still be made public.

**Do you want your identity to be public for this peer review?**  For information about this choice, including consent withdrawal, please see our Privacy Policy

Reviewer #1: No

Reviewer #2: **Yes:**  Monica Ewomazino Akokuwebe, PhD

**********

[NOTE: If reviewer comments were submitted as an attachment file, they will be attached to this email and accessible via the submission site. Please log into your account, locate the manuscript record, and check for the action link "View Attachments". If this link does not appear, there are no attachment files.]

To ensure your figures meet our technical requirements, please review our figure guidelines: https://journals.plos.org/plosone/s/figures

You may also use PLOS’s free figure tool, NAAS, to help you prepare publication quality figures: https://journals.plos.org/plosone/s/figures#loc-tools-for-figure-preparation.

NAAS will assess whether your figures meet our technical requirements by comparing each figure against our figure specifications.

Attachment Submitted filename: Reviewer Report_PLoS One.pdf

# Author comment: Author response to Decision Letter 1

**DOI:** 10.1371/journal.pone.0360475.r002

24 Apr 2026

Dear Editor,

Thank you for your comments and the detailed requirements. We have carefully addressed all the points as requested.

Specifically, the manuscript has been revised to meet PLOS ONE formatting and style requirements. The completed “Inclusivity in Global Research” questionnaire has been uploaded as Supporting Information. The minimal dataset required to replicate the study findings has been provided in the Supporting Information files. The ORCID iD for the corresponding author has been updated and validated in Editorial Manager. All Supporting Information files have been properly organized, and their captions have been added to the end of the manuscript with corresponding in-text citations updated accordingly. In addition, the text has been translated into English in accordance with requirements.

We hope that the revised manuscript meets the journal’s standards and look forward to your feedback.

Response to reviewer 1

Dear Reviewer,

Thank you for these valuable comments. We have carefully revised the manuscript accordingly, and the main revisions are summarized below.

1. We have now explicitly specified HbA1c as the primary endpoint in the revised manuscript. HbA1c was selected because it is the most widely accepted and clinically relevant indicator of long-term glycemic control in patients with diabetes and serves as a key target in diabetes management guidelines. In addition, HbA1c is closely associated with the risk of developing and progressing diabetic kidney disease. While renal outcomes such as UACR and eGFR are also clinically important, they were considered secondary endpoints to provide complementary information on renal function and disease progression. Given that HbA1c was pre-specified as the primary endpoint and the study was powered based on this outcome, analyses of secondary outcomes were considered exploratory, and the results were interpreted with caution due to multiple comparisons. These clarifications have been incorporated into the revised manuscript.

2. The GEE model specification has been further clarified by explicitly stating the use of a normal distribution with an identity link function, and robust standard errors were applied in all analyses. In addition, skewed variables (HbA1c and UACR) were log-transformed prior to analysis, and the results were back-transformed for ease of interpretation. These revisions have been incorporated into the Methods (Section 2.8) and reflected in the corresponding Results section.

3. We have conducted sensitivity analyses using multiple imputation with 10 imputed datasets to assess the robustness of our findings to missing data. The imputed datasets were analyzed using the same GEE models, and the results were pooled using Rubin’s rules implemented in R. The findings from the multiple imputation analyses were consistent with those from the primary analyses based on the original data, indicating that the conclusions are robust to the handling of missing data under the assumption of missing at random. These details have been added to the Methods and Results sections, and the full results are presented in Supplementary Table S5.

4. The sample size was determined based on the primary outcome. Given the repeated-measures design and the use of GEE, the analysis benefits from increased statistical efficiency by accounting for within-subject correlation over time. In addition, we have clarified the analytic details, including the covariates (education level, family history of diabetes, diabetes duration, and DKD risk score) and the use of an exchangeable working correlation structure. Finally, graphical displays of estimated marginal means with 95% confidence intervals have been added in the Supplementary Materials.

5. We have revised the Results section following a stepwise, decision-guided process. For outcomes with a statistically significant group-by-time interaction, we removed the reporting and interpretation of the main effects (time and group), as these are not meaningful in the presence of a significant interaction. Instead, we focused on the interaction term and presented time-specific comparisons. For outcomes without a significant interaction, the main effects were retained and interpreted appropriately.

Thank you again for your helpful suggestions.

Kind regards,

Response to reviewer 2

Dear Reviewer,

Thank you for your valuable comments. The manuscript has been revised accordingly. The Introduction has been updated to clarify the research gap and study objectives. The Methods section has been further detailed, including clarification of the sample size calculation and randomization procedures. The tables have been revised, and figures have been added to present the trends of each outcome. The Discussion has been improved to present the potential mechanisms of the self-management program. The intervention effectively improved glycemic regulation, emphasized the importance of glycemic control in preventing DKD progression, and highlighted participants’ susceptibility to DKD. By increasing awareness of personal risk, the intervention promoted behavioral change and strengthened self-management adherence, which may have contributed to the observed improvements in metabolic outcomes. In addition, study limitations and clinical implications have been described in Section 4.2. Finally, the manuscript has been revised according to the journal requirements, with language editing, standardized terminology, and improved consistency. In accordance with guideline usage, “kidney” is used for disease-related terms (e.g., diabetic kidney disease), while “renal” is used for functional descriptions (e.g., renal injury, renal inflammation).

Thank you again for your helpful suggestions.

Kind regards,

Attachment Submitted filename: Response to Reviewers.docx

# Peer review: Decision Letter 1

**DOI:** 10.1371/journal.pone.0360475.r003

**Authors:** Diego Moriconi

8 Jun 2026

Dear Dr.  Japar,

plosone@plos.org. When you're ready to submit your revision, log on to https://www.editorialmanager.com/pone/ and select the 'Submissions Needing Revision' folder to locate your manuscript file.

- A letter that responds to each point raised by the academic editor and reviewer(s). You should upload this letter as a separate file labeled 'Response to Reviewers'.
- A marked-up copy of your manuscript that highlights changes made to the original version. You should upload this as a separate file labeled 'Revised Manuscript with Track Changes'.
- An unmarked version of your revised paper without tracked changes. You should upload this as a separate file labeled 'Manuscript'.

If you would like to make changes to your financial disclosure, please include your updated statement in your cover letter. Guidelines for resubmitting your figure files are available below the reviewer comments at the end of this letter.

If applicable, we recommend that you deposit your laboratory protocols in protocols.io to enhance the reproducibility of your results. Protocols.io assigns your protocol its own identifier (DOI) so that it can be cited independently in the future. For instructions see: https://journals.plos.org/plosone/s/submission-guidelines#loc-laboratory-protocols. Additionally, PLOS ONE offers an option for publishing peer-reviewed Lab Protocol articles, which describe protocols hosted on protocols.io. Read more information on sharing protocols at https://plos.org/protocols?utm_medium=editorial-email&utm_source=authorletters&utm_campaign=protocols.

As the corresponding author, your ORCID iD is verified in the submission system and will appear in the published article. PLOS supports the use of ORCID, and we encourage all coauthors to register for an ORCID iD and use it as well. Please encourage your coauthors to verify their ORCID iD within the submission system before final acceptance, as unverified ORCID iDs will not appear in the published article. *Only*  the individual author can complete the verification step; PLOS staff *cannot*  verify ORCID iDs on behalf of authors.

We look forward to receiving your revised manuscript.

Kind regards,

Diego Moriconi

Academic Editor

PLOS One

Journal Requirements:

1. If the reviewer comments include a recommendation to cite specific previously published works, please review and evaluate these publications to determine whether they are relevant and should be cited. There is no requirement to cite these works unless the editor has indicated otherwise.

2. Please review your reference list to ensure that it is complete and correct. If you have cited papers that have been retracted, please include the rationale for doing so in the manuscript text, or remove these references and replace them with relevant current references. Any changes to the reference list should be mentioned in the rebuttal letter that accompanies your revised manuscript. If you need to cite a retracted article, indicate the article’s retracted status in the References list and also include a citation and full reference for the retraction notice.

Additional Editor Comments:

The study addresses a relevant research question and has the potential to make a valuable contribution to the field. Some issues regarding methodology, data interpretation, and presentation remain and would benefit from further clarification. These points appear readily addressable through revision, and I therefore recommend Minor Revision before further consideration.

[Note: HTML markup is below. Please do not edit.]

Reviewer's Responses to Questions

**Comments to the Author**

Reviewer #1: All comments have been addressed

Reviewer #2: All comments have been addressed

**********

2. Is the manuscript technically sound, and do the data support the conclusions?

Reviewer #1: (No Response)

Reviewer #2: Yes

**********

3. Has the statistical analysis been performed appropriately and rigorously?

Reviewer #1: (No Response)

Reviewer #2: Yes

**********

4. Have the authors made all data underlying the findings in their manuscript fully available?

The PLOS Data policy

Reviewer #1: (No Response)

Reviewer #2: Yes

**********

5. Is the manuscript presented in an intelligible fashion and written in standard English?

Reviewer #1: (No Response)

Reviewer #2: Yes

**********

Reviewer #1: (No Response)

Reviewer #2: Effectiveness of a Self-Management Intervention on Clinical Outcomes Among Patients with Type 2 Diabetes at High Risk of Diabetic Kidney Disease: A Randomized Controlled Trial

Peer Reviewer Report

1. Overall Assessment of the Research

This manuscript addresses an important clinical and public health issue by evaluating whether a structured self-management intervention can improve clinical outcomes among patients with type 2 diabetes mellitus who are at elevated risk of diabetic kidney disease. The study is highly relevant because diabetic kidney disease remains one of the most common and costly complications of diabetes globally, and preventive strategies targeted at high-risk individuals are urgently needed. The randomised controlled trial design strengthens the internal validity of the study and improves the reliability of causal inference. The findings contribute important evidence supporting early-stage behavioral and clinical management interventions before overt diabetic kidney disease develops. The manuscript is well structured and clinically meaningful. However, several areas require clarification and deeper analytical interpretation, particularly in the reporting of intervention components, outcome measurement, attrition handling, and interpretation of results. With revision, this manuscript would make a valuable contribution to diabetes care and chronic disease prevention literature.

2. Summary of the Entire Research

This study examined the effectiveness of a structured self-management intervention among adults with type 2 diabetes at high risk of diabetic kidney disease in a tertiary hospital in China. A two-arm randomised controlled trial was conducted with 134 participants assigned equally to intervention and control groups. The intervention group received a 12-week structured self-management programme followed by a 12-week follow-up, while the control group received standard care. Clinical outcomes including HbA1c, urinary albumin-to-creatinine ratio, estimated glomerular filtration rate, systolic blood pressure, LDL cholesterol, body mass index, and waist circumference were assessed at baseline, post-intervention, and follow-up. The intervention improved HbA1c, eGFR, systolic blood pressure, and LDL cholesterol compared with usual care. No significant improvements were observed for urinary albumin-to-creatinine ratio, body mass index, or waist circumference. The findings suggest that structured self-management may reduce modifiable risk factors associated with diabetic kidney disease progression and may contribute to delaying disease onset.

3. Major Comments

Research title

The title is clear, specific, and methodologically precise. It communicates the study population, intervention focus, outcome direction, and study design effectively. It is suitable for journal publication. The only possible refinement would be shortening slightly for readability, for example:

“Effectiveness of a Self-Management Intervention on Clinical Outcomes Among Patients with Type 2 Diabetes at High Risk for Diabetic Kidney Disease: A Randomised Controlled Trial.”

Abstract:

1. The abstract should clearly define what “high risk of diabetic kidney disease” means. What diagnostic or clinical criteria were used?

2. The intervention requires clearer description. What specific components were included such as education, counselling, monitoring, dietary modification, or medication adherence support?

3. The attrition numbers need correction and clarification because the reported “539 participants lost to follow-up” appears inconsistent.

4. The magnitude of effect should be reported. Include adjusted estimates, confidence intervals, or effect sizes rather than only stating significant improvement.

5. The conclusion should briefly note the practical implication for diabetes management and whether the intervention could be integrated into routine hospital care.

Introduction:

1. Does the introduction sufficiently describe the global and regional burden of diabetic kidney disease among people with type 2 diabetes?

2. Give us global prevalence of diabetic kidney disease and the issues that led to the low or high prevalence increase in high-income countries and Low-middle-income countries.

3. Can more evidence be added on why early intervention before clinically established diabetic kidney disease is especially important?

4. What is currently known about self-management interventions specifically among high-risk diabetic kidney disease populations?

5. How does this study differ from previous diabetes self-management trials?

6. Why was China selected as the study setting, and what contextual health system factors make this research important?

7. Are theoretical or behavioural frameworks guiding the intervention clearly explained?

8. Is the clinical importance of HbA1c, eGFR, and UACR sufficiently justified?

9. Can stronger justification be provided for targeting modifiable risk factors?

10. Is the research gap clearly articulated and distinct from general diabetes self-management literature?

11. Are the study objectives and hypotheses explicitly stated at the end of the introduction?

Methodology:

1. How was randomisation performed and who generated allocation?

2. Was allocation concealment used?

3. Were participants or assessors blinded?

4. How was sample size calculated and was power sufficient?

5. How was “high risk of diabetic kidney disease” operationalised?

6. What were inclusion and exclusion criteria?

7. What exactly did the intervention contain and how often was it delivered?

8. Was intervention fidelity monitored?

9. How was missing data handled beyond intention-to-treat analysis?

10. Was ethics approval and trial registration clearly reported?

Results and tables:

1. Correct participant flow numbers and ensure consistency.

2. Include a CONSORT flow diagram.

3. Present baseline demographic characteristics clearly in one table.

4. Report adjusted estimates with confidence intervals.

5. Clarify within-group and between-group comparisons.

6. Include exact p values where possible.

7. Present intervention adherence data.

8. Show outcome trends across T0, T1, and T2 clearly.

9. Indicate clinical significance, not only statistical significance.

10. Explain why UACR, BMI, and WC did not improve.

11. Ensure all tables use consistent decimal places and units.

12. Add supplementary sensitivity analysis outputs if journal permits.

Discussion:

1. Begin with the most important findings.

2. Explain why HbA1c improved.

3. Explain why eGFR improved despite short follow-up.

4. Interpret LDL and blood pressure improvements clinically.

5. Discuss why BMI and waist circumference did not improve.

6. Compare findings with prior randomized trials.

7. Discuss intervention feasibility in tertiary hospitals.

8. Consider whether effects may differ in primary care.

9. Address possible selection bias.

10. Discuss attrition implications.

11. Explain how findings support diabetic kidney disease prevention.

12. End with a balanced conclusion.

Strengths and Limitations

Strengths

The randomized controlled design strengthens causal inference. The study targets an understudied but clinically important high-risk population. Multiple objective clinical outcomes were measured. Intention-to-treat and sensitivity analyses improve methodological rigour. The follow-up period strengthens evidence on short-term sustainability.

Limitations

Single-site recruitment may reduce generalisability. Short follow-up limits long-term interpretation. Attrition requires clearer explanation. Lack of blinding may introduce bias. Behavioural adherence measures were not fully reported.

Study implications:

1. How can this intervention be integrated into routine diabetes clinics in China?

2. What are implications for preventing progression to diabetic kidney disease?

3. Could this intervention be adapted for broader community-based diabetes care?

Future research directions:

1. Can effects be sustained beyond six months?

2. Would similar outcomes be observed in multicenter studies?

3. Which intervention components are most effective?

4. What is the cost-effectiveness of implementation?

Reference section:

1. Are all recent diabetic kidney disease prevention trials cited?

2. Are references consistently formatted?

3. Are foundational diabetes self-management guidelines included?

Proofreading and editing recommendations

1. Correct typographical and numerical inconsistencies.

2. Improve consistency of terminology across manuscript.

3. Tighten sentence structure for clarity and flow.

Recommended articles for strengthening references

1. Zimbudzi, E., Lo, C., Misso, M. L., Ranasinha, S., Kerr, P. G., Teede, H. J., & Zoungas, S. (2018). Effectiveness of self-management support interventions for people with comorbid diabetes and chronic kidney disease: A systematic review and meta-analysis. Systematic Reviews, 7, 84. https://doi.org/10.1186/s13643-018-0748-z

2. Waki, K., Nara, M., Enomoto, S. et al. Effectiveness of DialBetesPlus, a self-management support system for diabetic kidney disease: Randomized controlled trial. npj Digit. Med. 7, 104 (2024). https://doi.org/10.1038/s41746-024-01114-8

3. Khavere, S., Hadjiconstantinou, M., Miksza, J., Hagan, J., Naderpour, S., Hassen, S. N., Karimi, Z., & Gillies, C. L. (2025). Effectiveness of self‐management interventions on Type 2 diabetes among young adults (18–45 years): A systematic review and meta‐analysis. Diabetic Medicine, 42(10), e70127. https://doi.org/10.1111/dme.70127

4. Gathu, Catherine W., Shabani, Jacob, Kunyiha, Nancy, & Ratansi, Riaz. (2018). Effect of diabetes self-management education on glycaemic control among type 2 diabetic patients at a family medicine clinic in Kenya: A randomised controlled trial. African Journal of Primary Health Care & Family Medicine , 10(1), 1-9. https://doi.org/10.4102/phcfm.v10i1.1762

5. Edward Zimbudzi, Clement Lo, Sanjeeva Ranasinha, Peter G. Kerr, Timothy Usherwood, Alan Cass, Gregory R. Fulcher, Sophia Zoungas (2017). Self-management in patients with diabetes and chronic kidney disease is associated with incremental benefit in HRQOL. Journal of Diabetes and its Complications, Volume 31, Issue 2. Pages 427-432. ISSN 1056-8727, https://doi.org/10.1016/j.jdiacomp.2016.10.027. (https://www. sciencedirect.com/science/article/pii/S1056872716303920)

Overall, this is a valuable and clinically relevant manuscript. The trial design and focus on early prevention are strong. With clarification of intervention details, correction of participant flow reporting, and deeper interpretation of findings, the manuscript would be substantially strengthened and suitable for journal publication.

**********

what does this mean?). If published, this will include your full peer review and any attached files.

If you choose “no”, your identity will remain anonymous but your review may still be made public.

**Do you want your identity to be public for this peer review?**  For information about this choice, including consent withdrawal, please see our Privacy Policy

Reviewer #1: No

Reviewer #2: **Yes:**  Monica Ewomazino Akokuwebe, PhD

**********

[NOTE: If reviewer comments were submitted as an attachment file, they will be attached to this email and accessible via the submission site. Please log into your account, locate the manuscript record, and check for the action link "View Attachments". If this link does not appear, there are no attachment files.]

To ensure your figures meet our technical requirements, please review our figure guidelines: https://journals.plos.org/plosone/s/figures

You may also use PLOS’s free figure tool, NAAS, to help you prepare publication quality figures: https://journals.plos.org/plosone/s/figures#loc-tools-for-figure-preparation.

NAAS will assess whether your figures meet our technical requirements by comparing each figure against our figure specifications.

Attachment Submitted filename: Peer Reviewer 2.pdf

# Author comment: Author response to Decision Letter 2

**DOI:** 10.1371/journal.pone.0360475.r004

29 Jun 2026

Dear Editor and Reviewers,

We sincerely thank you for your valuable comments. We carefully reviewed all the comments and revised the manuscript where appropriate. Detailed responses are provided below.

Abstract： Thank you for your valuable comments. We revised the Abstract to clarify the definition of high-risk DKD, improve the description of the intervention, correct the participant attrition information, and strengthen the practical implications in the Conclusion. Considering the Abstract word limit, detailed statistical results remain presented in the Results section.

Introduction：Thank you for your valuable comments. We carefully reviewed the Introduction and confirmed that the current version already addresses the disease burden, rationale for early intervention, existing evidence, research gap, and study objectives. Therefore, the current text has been retained.

Methods：Thank you for your valuable comments. We carefully reviewed the Methods section and confirmed that the current manuscript already provides sufficient information regarding the study design, randomization, blinding, sample size calculation, participant eligibility, intervention, missing data handling, ethical approval, and trial registration. Therefore, the current text has been retained.

Results and Tables：Thank you for your valuable comments. We carefully reviewed the Results and tables. The participant flow numbers were verified for consistency, and the CONSORT flow diagram is provided as a separate uploaded file. Baseline characteristics are presented in Table 3, and adjusted estimates, 95% confidence intervals, and within-group and between-group comparisons are presented in Table 4. Statistical values and p values are consistently reported to three decimal places, and detailed sensitivity analysis results are provided in Supplementary File S5.

Discussion： We carefully reviewed the Discussion and confirmed that the current manuscript already addresses the main findings, their interpretation, comparisons with previous studies, and the implications for DKD prevention. We revised the Implications for Clinical Practice section to further emphasize the feasibility and scalability of the video-based intervention and revised the Conclusion to provide a more balanced summary of the study findings.

Attachment Submitted filename: Response_to_Reviewers_auresp_2.docx

# Editor report: Decision Letter 2

**DOI:** 10.1371/journal.pone.0360475.r005

**Authors:** Diego Moriconi

27 Aug 2026

Effectiveness of a Self-Management Intervention on Clinical Outcomes Among Patients With Type 2 Diabetes at High Risk for Diabetic Kidney Disease: A Randomized Controlled Trial

PONE-D-25-66443R2

Dear Dr. Salimah Japar

We’re pleased to inform you that your manuscript has been judged scientifically suitable for publication and will be formally accepted for publication once it meets all outstanding technical requirements.

Within one week, you’ll receive an e-mail detailing the required amendments. When these have been addressed, you’ll receive a formal acceptance letter and your manuscript will be scheduled for publication.

An invoice will be generated when your article is formally accepted. Please note, if your institution has a publishing partnership with PLOS and your article meets the relevant criteria, all or part of your publication costs will be covered. Please make sure your user information is up-to-date by logging into Editorial Manager at Editorial Manager® and clicking the ‘Update My Information' link at the top of the page. For questions related to billing, please contact billing support.

If your institution or institutions have a press office, please notify them about your upcoming paper to help maximize its impact. If they’ll be preparing press materials, please inform our press team as soon as possible -- no later than 48 hours after receiving the formal acceptance. Your manuscript will remain under strict press embargo until 2 pm Eastern Time on the date of publication. For more information, please contact onepress@plos.org.

Kind regards,

Diego Moriconi

Academic Editor

PLOS One

Additional Editor Comments (optional):

The manuscript has substantially improved following revision, and the authors have adequately addressed the reviewer comments.

I therefore recommend acceptance for publication.

Reviewers' comments:

# Editor report: Acceptance letter

**DOI:** 10.1371/journal.pone.0360475.r006

**Authors:** Diego Moriconi

PONE-D-25-66443R2

PLOS One

Dear Dr. Japar,

I'm pleased to inform you that your manuscript has been deemed suitable for publication in PLOS One. Congratulations! Your manuscript is now being handed over to our production team.

At this stage, our production department will prepare your paper for publication. This includes ensuring the following:

* All references, tables, and figures are properly cited

* All relevant supporting information is included in the manuscript submission,

* There are no issues that prevent the paper from being properly typeset

You will receive further instructions from the production team, including instructions on how to review your proof when it is ready. Please keep in mind that we are working through a large volume of accepted articles, so please give us a few days to review your paper and let you know the next and final steps.

Lastly, if your institution or institutions have a press office, please let them know about your upcoming paper now to help maximize its impact. If they'll be preparing press materials, please inform our press team within the next 48 hours. Your manuscript will remain under strict press embargo until 2 pm Eastern Time on the date of publication. For more information, please contact onepress@plos.org.

You will receive an invoice from PLOS for your publication fee after your manuscript has reached the completed accept phase. If you receive an email requesting payment before acceptance or for any other service, this may be a phishing scheme. Learn how to identify phishing emails and protect your accounts at https://explore.plos.org/phishing.

If we can help with anything else, please email us at customercare@plos.org.

Thank you for submitting your work to PLOS One and supporting open access.

Kind regards,

PLOS One Editorial Office Staff

on behalf of

Dr. Diego Moriconi

Academic Editor

PLOS One
